# Interstitial Oxide Ion Order and Conductivity in La_1.64_Ca_0.36_Ga_3_O_7.32_ Melilite**

**DOI:** 10.1002/anie.200906220

**Published:** 2010-02-28

**Authors:** Man-Rong Li, Xiaojun Kuang, Samantha Y Chong, Zhongling Xu, Christopher I Thomas, Hongjun Niu, John B Claridge, Matthew J Rosseinsky

**Affiliations:** * Department of Chemistry, University of Liverpool, LiverpoolL69 7ZD (UK), Fax: (+44) 151-794-3598

**Keywords:** defect structures, ion conductivity, oxido ligands, solid-state structures

Solid oxide fuel cells (SOFCs) are a major candidate technology for clean energy conversion because of their high efficiency and fuel flexibility.^1^ The development of intermediate-temperature (500–750 °C) SOFCs requires electrolytes with high oxide ion conductivity (exceeding 10^−2^ S cm^−1^ assuming an electrolyte thickness of 15 μm^1^). This conductivity, in turn, necessitates enhanced understanding of the mechanisms of oxide ion charge carrier creation and mobility at an atomic level. The charge carriers are most commonly oxygen vacancies in fluorites^2^, ^3^ and perovskites.^3^, ^4^ There are fewer examples of interstitial-oxygen-based conductors such as the apatites^5^, ^6^ and La_2_Mo_2_O_9_-based materials,^7^–^9^ so information on how these excess anion defects are accommodated and the factors controlling their mobility is important.

The A_2_B_3_O_7_ melilite structure consists of anionic layers of five-membered rings of two totally condensed (four neighboring tetrahedra linked by B-O-B bonds) and three partially condensed (three neighboring tetrahedra) BO_4_ tetrahedra, separated by sheets of A cations located above the five-ring centers (Figure 1 a, and Figure S1.1 in the Supporting Information). Previously,^10^ we demonstrated that A-site substitution in melilite LaSrGa_3_O_7_ (A_2_=LaSr, B=Ga) affords La_1.54_Sr_0.46_Ga_3_O_7.27_, in which interstitial oxygen atoms (O_int_) are located in the five-rings of the two-dimensional tetrahedral network, gives pure oxide ion conductivity of 0.02–0.1 S cm^−1^ over the 600–900 °C temperature range.^11^ LaCaGa_3_O_7_ also adopts the tetragonal melilite structure (space group *P*42_1_*m*) found for the Sr phase.^12^, ^13^ When doped to introduce oxide charge carriers in the La_1+*x*_Ca_1−*x*_Ga_3_O_7+0.5*x*_ series, the tetragonal structure is retained to *x*=0.5. Herein, we address the impact on the physical properties of a new lower-symmetry phase formed to accommodate the higher interstitial doping levels 0.55≤*x*≤0.64.

**Figure 1:**
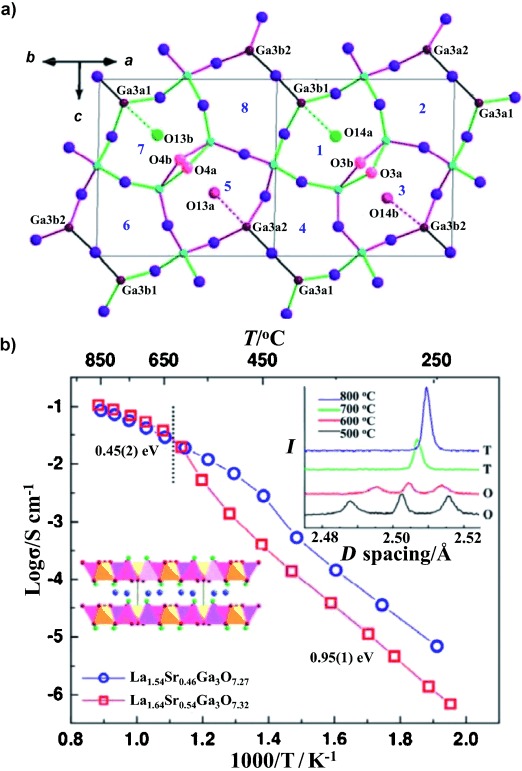
a) View of the average structure of the Ga_3_O_7+*x*/2_ network along the [110] direction for La_1.64_Ca_0.36_Ga_3_O_7.32_. Four of the eight possible oxygen interstitial sites (Ch*n*, *n*=1–8, labeled in blue) in the five-rings are occupied (Ch1 (O14a) 58.1(1) %, Ch3 (O14b) 23.9(1) %, Ch5 (O13a) 28.0(1) %, Ch7 (O13b) 17.9(1) %). The local ordering involves coupled occupancy of the Ch1/Ch7 (green) and Ch5/Ch3 (red) sites, which differ in the polarity of the interstitial displacement along the [101] direction towards the single three-connected GaO_4_ unit within each ring, as indicated with broken lines of different colors. The two orientations of the three-connected Ga_2_O_7_ dimers within the five-rings are shown with black Ga-O-Ga bonds (unoccupied ring centroids) and by representing the bridging oxygen atoms (O3a/O3b, O4a/O4b) of the centroid-occupied rings in pink. b) Arrhenius plot of total conductivity for La_1.64_Ca_0.36_Ga_3_O_7.32_ compared with that for La_1.54_Sr_0.46_Ga_3_O_7.27_. The dashed line marks the phase transition shown by variable-temperature NPD (top-right inset) from O (orthorhombic) to T (tetragonal); the bottom-left inset shows the stacking of La_1+*x*_Ca_1−*x*_ (blue) and Ga_3_O_7_ layers in melilite.

X-ray powder diffraction from La_1.64_Ca_0.36_Ga_3_O_7.32_ (Section 2 in the Supporting Information) indicates that the lowering of symmetry at high doping levels occurs by formation of an orthorhombic cell with expansion of the *ab* plane containing the B_3_O_7_ network, consistent with the *Cmm2* subgroup of *P*42_1_*m* (*a*≍11.416, *b*≍11.226, *c*≍5.248 Å, where *a*≍*b*≍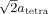, *c*=*c*_tetra_). Selected area electron diffraction and high-resolution TEM imaging require further doubling of *c* to give a body-centered supercell with no other systematic absences (Figure S3.2-3 in the Supporting Information), consistent with space groups *Imm*2, *Im*, and *I*1.

Variable-temperature neutron powder diffraction (NPD; Figure 1 b (top-right inset) and Figure S4.1 in the Supporting Information) shows a transition between the low-temperature orthorhombic and the small-cell tetragonal structure between 600 and 700 °C, corresponding to a strong anomaly in the temperature dependence of the conductivity around 650 °C (Figure 1 b). At this point, the activation energy changes from 0.95(1) eV at low temperature to 0.45(2) eV at high temperature. The ionic conductivity of the distorted phase is lower than that of the less doped La_1.54_Sr_0.46_Ga_3_O_7.27_, which retains the small-cell tetragonal structure at all temperatures, thus suggesting that the structural phase transition is produced by static ordering of the interstitial oxide charge carriers.

Rietveld analysis of the NPD data showed that the best fits were obtained in *P*1 (*a*=9.5736 (1), *b*=9.5748(1), *c*=9.5692(1) Å, *α*=106.78(1), *β*=108.17(1), *γ*=113.51(1)°, *V*=672.85(1) Å^3^; Figure S3.1 in the Supporting Information shows the relationship to the *I*1 cell). There are eight inequivalent pentagonal channels that are candidate interstitial sites in the *P*1 cell, rather than the single site in the disordered tetragonal structure. Conventional least-squares refinements and simulated annealing analysis reveal the ordering of the interstitial oxide charge carriers over these sites that produces the conductivity reduction. The interstitial content was constrained as O_7.32_ per formula unit, with all eight sites equally occupied and random La/Ca site occupancy in the starting model. Refinement converged to *R_w_*_p_/*R*_p_=3.21/4.60 %, *χ*^2^=4.28 (Figure S5.6 and Table S5.4-5 in the Supporting Information). The interstitial oxygen atoms occupy four of the eight pentagonal channels (Ch*n*, *n*=1–8 in Figure 1 a). Random occupancy of the interstitial sites gives a poorer fit (*χ*^2^=5.27). There is partial cation ordering on the A sites: half are fully occupied by La (La1a, La2a, La2b, and La3a), while the others are La/Ca mixed sites (La/Ca1b, La/Ca3b, La/Ca4a, and La/Ca4b), of which only La/Ca3b and La/Ca4a have significantly more than statistical amounts of calcium (Figure S5.7 and Table S5.4 in the Supporting Information). The simulated annealing analysis, which is less susceptible to false minima,^14^ gives a qualitatively identical and quantitatively similar description of the A site and interstitial oxygen ordering (Section 6 in the Supporting Information).

Although the refined average structure defines the O interstitial and La dopant occupancies relative to the symmetry-distinct centers of the five-rings of the Ga_3_O_7_ layer, a more chemically acceptable locally relaxed model is needed to give quantitatively reasonable bond valence sums (BVS)^15^, ^16^ at the under-bonded interstitial oxide sites and at the isolated three-connected GaO_4_ tetrahedra that are closest to the interstitial oxygen atoms (BVS≍3.30, Table S5.5 in the Supporting Information). A split model for relaxation around the defects was refined (*χ*^2^=4.81; Figure 2 and Figure S7.1 and Table S7.1-3 in the Supporting Information), with two sets of positions representing the resulting bulk (no interstitial oxide present, tetrahedral Ga) and defect (five-coordinate Ga) structural environment around the interstitial oxygen atoms. The displacements of gallium and oxygen atoms from the bulk to the defect structure are shown in Figure 2, Figure 3, and Figure S7.2-4 in the Supporting Information. We use the most highly occupied interstitial site O14a (average structure BVS=1.28) to show how synergic bonding geometry changes at the isolated three-connected Ga3b1 site and A counterion displacement towards O14a accommodate the excess oxide ions.

**Figure 2:**
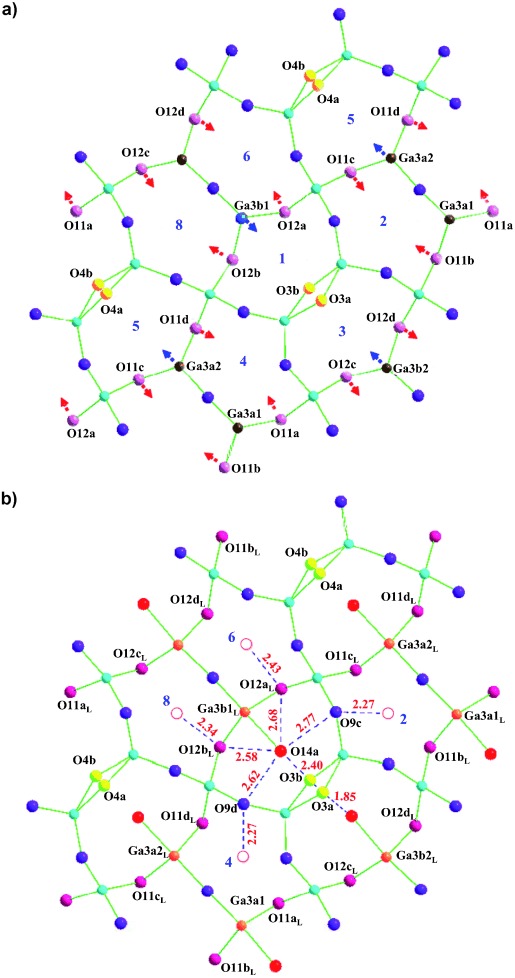
The projection along [110] of a) bulk and b) defect structure around the most highly occupied five-ring centroid Ch1. To accommodate the interstitial oxide O14a, O12a and O12b are displaced to sites O12a_L_ and O12b_L_ toward neighboring rings Ch6 and Ch8 (marked with arrows in (a)). The occupancy of O3a when O14a is present locally makes Ch3 the most distorted of all the five rings sharing edges with Ch1. Distances from the bridge oxides defining the five-ring around Ch1/O14a to the centroids of neighboring channels are marked in red in (b), in which the centroids of unoccupied channels are indicated as open circles.

The two A sites above and below the Ch1 five-ring displace towards O14a, with the pure La site closer than the mixed site (La/Ca4a_L_−O14a=2.51(1) Å, La3a_L_−O14a=2.46(1) Å) to electrostatically stabilize the interstitial oxide (Figure S7.2 in the Supporting Information). The displacement of the defect model position Ga3b1_L_ (0.17(1) Å) from the tetrahedron center Ga3b1 in the bulk model towards O14a into a triangular face of the original tetrahedron (Figure 3 a) shortens this gallium–oxygen bond from 2.177(6) Å to 2.305(4) Å. Oxygen atom O14a thus enters the coordination environment of Ga3b1_L_ at one of the axial positions of the resulting trigonal bipyramidal Ga3b1_L_O_5_ unit (Figure 3 b). It should be emphasized that when the other four Ga centers defining this five-ring are allowed to relax by a similar site splitting, they move slightly away from O14a rather than towards it, thus demonstrating that only the isolated three-connected Ga center binds to the interstitial oxide. The interstitial atom O14a makes close contacts (ca. 1.9–2.5 Å) to three other oxygen atoms (O3b, O12a, and O12b) in the five-membered ring; the distances to O9c and O9d are longer (2.62(1) and 2.77(1) Å, Figure 2 b). The resulting displacement of 0.56(1) Å for O12a_L_ and of 0.49(1) Å for O12b_L_ bound to Ga3b1_L_ increases the mean O-Ga3b1_L_-O angles in the equatorial plane to 119.4(2)° (from 105.2(2)° in the average structure) and makes the mean O_apical_-Ga3b1_L_-O_equatorial_ angle 89.7(2)° (Table S7.2-3 in the Supporting Information), while the mean O-Ga3b1-O angle in the bulk GaO_4_ tetrahedron is 108.9(1)°. These two bridging oxygen atoms, and also O3a, which bridges the Ga_2_O_7_ dimer of three-connected centers forming the pentagon edge opposite Ga3b1, are now 2.40(1)–2.70(1) Å from O14a and as a result are displaced towards the centers of neighboring pentagonal channel (Ch3, Ch6, and Ch8 in Figure 2 a). This expansion of Ch1 around O14a results in much shorter distances from its edge-bridging oxygen atoms to the centroids of the five neighboring edge-sharing channels than to O14a (Figure 2 b). This model decreases the BVS of Ga3b1 to 2.91 (Ga3b1O_4_ tetrahedron) and 2.97 (Ga3b1_L_O_5_ trigonal bipyramid) and increases the O14a BVS to 1.91, thus providing a satisfactory description of the bonding around the defect.

**Figure 3:**
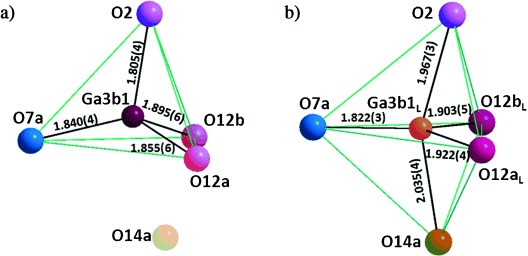
Structural relaxation around the most highly occupied interstitial site O14a. Ga3b1_L_, O12a_L_, O12b_L_, and O3a are involved in the local structural relaxation. a) Tetrahedral GaO_4_ in the absence of O14a. b) GaO_5_ trigonal bipyramid formed by O14a. O7a is the nonbridging terminal oxygen atom.

This relaxation around O14a prevents interstitial occupancy of any of the five nearest-neighbor ring centers to Ch1 (Ch2, Ch3, Ch4, Ch6, and Ch8; Figure 2 a). The doping dependence of the structure can be rationalized by consideration of the longer-range interaction of these distortions. Each interstitial site is surrounded by seven nearest-neighbor interstitial sites (Figure 4), five sharing a common edge and the more distant two sharing a fully condensed tetrahedral GaO_4_ vertex. The closest of the five edge-sharing neighbors (Ch3 in the case of the Ch1 occupancy discussed above—this is the ring that is most distorted by the presence of the interstitial oxide in the defect model) shares a Ga_2_O_7_ unit of two bridged three-connected GaO_4_ tetrahedra, while the four next-nearest neighbors share edges linking three- and four-connected tetrahedra.

**Figure 4:**
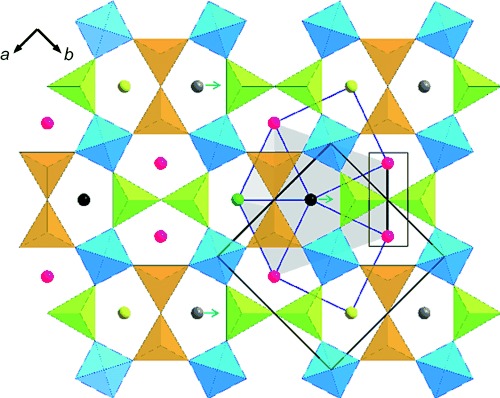
The seven five-ring centroids surrounding an occupied interstitial site (black sphere). Red spheres represent rings sharing an edge of 3- and 4-connected GaO_4_ units; green spheres denote a 3-connected Ga_2_O_7_ edge (this ring is the most heavily distorted by the presence of the interstitial oxide); yellow spheres show rings linked by corner sharing; 4-connected GaO_4_ units are colored cyan. The two orientations of the Ga_2_O_7_ dimers of 3-connected tetrahedra are shown in green (black bonds in Figure 1 a) and orange (pink bridging oxygen atoms in Figure 1 a) and represent the two displacement directions of the interstitial oxide ions in the disordered tetragonal structure. The green arrow shows the single displacement direction in the ordered orthorhombic structure. The five nearest-neighbor interstitial sites are represented as a gray pentagon centered on the occupied ring. The gray spheres represent a second set of sites whose occupancy does not share the most distorted near-neighbor ring (green spheres) of the first set. The highlighted black line connects two ring centroids (marked as black spheres in Figure 5 b), which can only have two of the three neighboring black and gray sites occupied at the observed composition.

The observed supercell has eight crystallographically distinct centroids as candidate oxygen interstitial sites, and sole occupancy of one of these sets at 1/8 (12.5 %) coverage (*x*=0.5) would leave all seven of the rings neighboring the occupied interstitial site empty, with none of the nearest neighbors of these rings occupied (Figure 4). This situation would allow relaxation of the five nearest-neighbor edge-sharing rings around an interstitial oxide (Figure 2) to take place without interference from similar distortions enforced by the nearest occupied interstitial sites; only the more distant corner-sharing empty nearest neighbors are shared by the occupied sites.

In fact, the situation is more complex, as the interstitial oxide ions remain positionally disordered in the tetragonal structure (in which all five-ring centroid sites are equivalent) beyond 12.5 % coverage to 13.75 % (*x*=0.55). Sharing of the most distorted five edge-sharing neighbors can be avoided in disordered interstitial occupancies within the tetragonal structure up to *x*>0.5 (Figure 5 a). The observation of long-range order of the displacements between *x*=0.5 and 0.6 suggests that the sharing of the empty corner-shared neighbors by the occupied interstitial sites does have some energetic significance.

**Figure 5:**
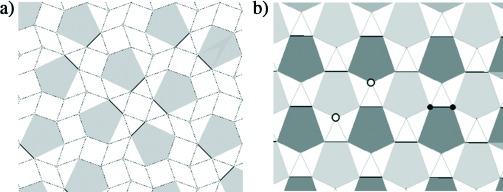
a) Disordered interstitial occupancy less than 1/8 in the tetragonal structure, represented using the pentagon of five neighbors (Figure S8.1 in the Supporting Information) of an occupied sphere from Figure 4. The pentagon edges highlighted in black connect the two five-ring centroids neighboring the occupied interstitial site that share a common three-connected Ga_2_O_7_ dimer and indicate the two orientations of interstitial displacement possible in the tetragonal structure, in contrast to the distorted structure (Figure 1), in which only one displacement direction is present. b) The maximally doped ordered interstitial structure (pentagon centroids represent occupied sites, vertices are empty ones) in which the two of the eight sites which share a common interstitial displacement sense and direction are occupied. This situation avoids two interstitial sites sharing the most distorted unoccupied ring (white spheres) as a near neighbor. Dark spheres represent two ring centroids sharing a common three-connected Ga_2_O_7_ edge, equivalent to the highlighted spheres connected by a black line in Figure 4.

At *x*=0.55, interstitial ordering occurs to minimize the overlap of the strain fields produced by the displacements into neighboring rings. This ordering can be discussed in terms of the direction of the displacement of the interstitial oxide towards the isolated 3-connected GaO_4_ unit within each ring, away from the edge defined by the Ga_2_O_7_ unit formed by the bridged 3-connecting GaO_4_ units. In the disordered tetragonal structure, there are two equivalent orientations of this edge (Figure 4 and Figure 5 a), so both displacement directions occur. In order to ensure that the most distorted neighboring ring to an occupied interstitial site (sharing the Ga_2_O_7_-bridged edge away from which O_int_ displaces to bond with the isolated 3-connected Ga atom) is not shared, it is necessary to order the occupancy of the interstitial sites at *x*>0.6 by selecting one of these two displacement directions that are degenerate in the tetragonal structure. This stipulation requires four of the eight interstitial sites corresponding to the other displacement direction to be empty. It is then possible to select a second site from within the remaining four that has the same polarity of displacement along the unique direction as the first and will leave empty all seven nearest neighbors of both occupied sites (Figure 5 b). This arrangement leaves empty all four of the remaining edge-sharing rings around the most distorted ring produced by both sets of interstitial oxides (Ch3 into which O3a displaces in the case of the O14a interstitial, represented as white spheres in Figure 5 b). This situation avoids sharing of the major distortions produced by the two occupied interstitial sites. The other four edge-sharing rings around the first interstitial site share one of their four less-distorted edges with the second occupied sublattice (Figure 5 b).

Full occupancy of these two sites sharing the same direction and sense of displacement would give 25 % coverage (*x*=1). The real situation is more complex, as all four of the sites corresponding to one displacement direction (and thus to both senses of this displacement shown in Figure 1) are partly occupied. The observed composition can be rationalized in terms of minimizing the strain in two next-most-distorted rings neighboring an occupied interstitial site (Ch6 and Ch8 in the case of Ch1), which are bridged by a common Ga_2_O_7_ dimer (Figure 4 and Figure 5 b) either randomly or in an ordered manner. The Ch1 (58 %) and Ch7 (18 %) sites correspond to two arrays with the same direction and sense of displacement (green dotted lines in Figure 1), with the O14a Ch1 occupancy locally allowing only approximately 1/3 of the Ch7 sites to be occupied, rather than all of them, to minimize the strain associated with this empty near-neighbor ring sharing and give a total 2/3 occupancy of the possible sites. This is an “ordered” model in which one of the eight sites is fully occupied and the second site that shares the same sense and direction of the displacement is 1/3 occupied. The occupancy of the Ch3/Ch5 pair at 24/28 % corresponds to the remaining pair of the four centroids sharing the same displacement direction, but with the sense of the displacement along that direction being opposed to the ordered pair (pink dotted lines in Figure 1); as the occupancy of both sites is approximately equal, this situation corresponds to disordered 2/3 occupancy of both sites. As less than 40 % of sites in total are occupied, this arrangement can occur in regions not neighboring the ordered regions referred to above. Simultaneous local occupancy of the Ch1/Ch7 and Ch3/Ch5 pairs would place interstitial oxides as nearest neighbors and would thus be disfavored, as relaxation into the rings neighboring an interstitial site would be suppressed.

The refined structure of the interstitial-ordered orthorhombic phase reveals how the distortions required to stabilize the interstitial oxides at both the tetrahedral and the larger A sites become coupled at high interstitial densities to prevent unfavorable local interactions between carriers. Although the refined cell would need to be doubled again to allow for total carrier order, thus explaining the rather high residual ionic conductivities, the effect on interstitial mobility of the need to rearrange the complex relaxation around a mobile carrier without adversely affecting potential neighbors in the surrounding five-rings can be gauged by the extent of the displacements required between the interstitial-containing and interstitial-free parts of the superstructure, and the requirement to order the La dopants on the A site around the interstitial oxides.^17^

## Supporting Information

Detailed facts of importance to specialist readers are published as ”Supporting Information”. Such documents are peer-reviewed, but not copy-edited or typeset. They are made available as submitted by the authors.
