# Supplementary material for: Interstitial Oxide Ion Order and Conductivity in La1.64Ca0.36Ga3O7.32 Melilite
Source: Angew Chem Int Ed Engl. 2010 Feb 28;49(13):2362–6. doi: 10.1002/anie.200906220 (PMC3430004; doi:10.1002/anie.200906220)
Supplement: Supplementary file 1 [file anie0049-2362-SD1.pdf]

Angewandte Chemie
Eine Zeitschrift der Gesellschaft Deutscher Chemiker

Supporting Information

© Wiley-VCH 2010

69451 Weinheim, Germany

Interstitial Oxide Ion Order and Conductivity in $La_{1.64}Ca_{0.36}Ga_3O_{7.32}$ Melilite**

Man-Rong Li, Xiaojun Kuang, Samantha Y. Chong, Zhongling Xu, Christopher I. Thomas, Hongjun Niu, John B. Claridge, and Matthew J. Rosseinsky**

anie_200906220_sm_miscellaneous_information.pdf

The supporting information is organised as follows:

1. Experimental Section

2. XRD data for $La_{1+x}Ca_{1-x}Ga_3O_{7+0.5x}$

3. SAED and HRTEM analysis

4. Phase transition (VT NPD)

5. Structure refinement for $La_{1.64}Ca_{0.36}Ga_3O_{7.32}$ in average models

6. Simulated annealing approach

7. Structure refinement for $La_{1.64}Ca_{0.36}Ga_3O_{7.32}$ in split model

8. Interstitial oxide and A-site cation ordering--composition and crystal structure

1. Experimental Section

The $A_2B_3O_7$ melilite structure, where A is a large eight-coordinated cation (A = Ca, Sr, Na, Ba, La) and B is small cation (B = Mg, Zn, Al, Ga, Si, Ge, …) in tetrahedral coordination, consists of anionic layers of five-membered rings of totally (four neighbouring tetrahedra) and partially condensed (three neighbouring tetrahedral with a terminal oxygen) BO_4 tetrahedra, separated by parallel sheets of A cations located over the five-ring centres along [001] (Figure S1.1).

$La_{1+x}Ca_{1-x}Ga_3O_{7+\delta}$ ($0 \leq x \leq 0.7$) (A = La/Ca, B = Ga) were prepared by solid state reaction using La_2O_3 (99.999%, Alfa Aesar), $CaCO_3$ (99.997%, Alfa Aesar) and Ga_2O_3 (99.999%, Alfa Aesar) as starting materials, which were mixed in ethanol and calcined at 1200 °C for 12 h in alumina crucibles. After regrinding, the powders were uniaxially pressed into pellets and fired at 1400 °C for samples with x < 0.5 and 1350 °C for samples with x > 0.5 for 12 h at 5 °C/min heating and cooling rates. To compensate the Ga volatilization during the reaction which results in the form of the second phase Ca-doped $LaGaO_3$, a slight excess of Ga_2O_3 (~0-1.7 mol% for x ≤ 0.6; ~2-3.3mol% for 0.6 < x ≤ 0.65; ~5mol% for x > 0.65) was added into the initial starting mixture. Attempts to obtain single phase materials at $x = 0.65$ by adding excess Ga_2O_3 (e.g. ~ 2 mol% and ~ 3.3 mol%) failed to remove the second phase Ca-doped $LaGaO_3$. Slightly reducing the La/Ca ratio to $x = 0.64$ with ~ 2.3 mol% extra Ga_2O_3 (i.e. initial composition $La_{1.64}Ca_{0.36}Ga_{3.07}$) produced a nearly single phase material $La_{1.64}Ca_{0.36}Ga_3O_{7.32}$ with a very small Ga_2O_3 impurity. To avoid reacting with the alumina, the pellets were put on platinum foil during the preparation. The phase purity was checked by powder X-ray diffraction data with a Panalytical X'pert Pro Multi-Purpose X-ray diffractometer (Co $K\alpha_1$ radiation λ = 1.78901Å). Silicon was added as an internal standard during the laboratory XRD experiments in order to refine the variation of the cell parameter with composition. Time-of-flight (TOF) neutron powder diffraction (NPD) data of $La_{1.64}Ca_{0.36}Ga_3O_{7.32}$ sample were collected from ambient temperature to 800 °C on the HRPD diffractometer at ISIS. After the variable temperature diffraction, the sample

was offline cooled to ambient temperature and another ambient temperature TOF data was collected from comparison with that for the as-made sample. Rietveld refinement was carried out using the GSAS package[1]. Compositional analysis was carried out by using the EDAX analyzer on a JEOL 2000FX transmission electron microscope operated at 200 KV. Ac Impedance Spectroscopy (IS) measurements in air from 200 °C to 850 °C were performed with a Solartron 1255B Frequency

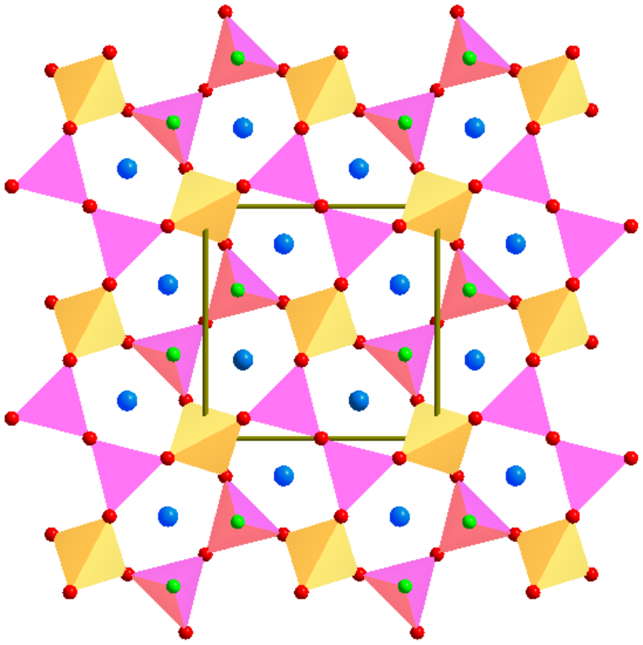

(a)

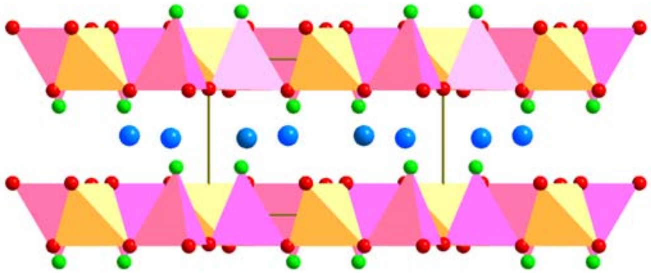

(b)

Figure S1.1. Slab view of $A_2B_3O_7$ melilite structure along (a) [001] and (b) [010] direction, showing the five membered BO_4 rings and location of A cations. Four connected BO_4 tetrahedron, yellow; three connected BO_4, purple; A cation, blue; bridging oxygen, red; terminal oxygen, green.

Response Analyzer, a Solartron 1296 dielectric interface and a Solartron 1287 electrochemical interface over the 10^{-2}-10^{6} Hz frequency range. Prior to the measurement, platinum paste was painted on the opposite faces of the pellets and fired at 800 °C for 30 minutes in air.

2. XRD data for $La_{1+x}Ca_{1-x}Ga_3O_{7+0.5x}$

The parent material $LaCaGa_3O_7$ crystallizes in a tetragonal melilite structure in $P\bar{4}2_1m$ (a = 7.9553(2) Å, c = 5.2727(2) Å). With the substitution of Ca^{2+} by La^{3+}, the tetragonal phase extends to $La_{1.5}Ca_{0.5}Ga_3O_{7.25}$. The cell parameters of $La_{1+x}Ca_{1-x}Ga_3O_{7+0.5x}$ obey Vegard's law for $0 < x < 0.5$. The La^{3+} substitution expanded the a-axis and shortened the c-axis. The volume increased with increasing La^{3+} content. Each (hkl) (h and $k \neq 0$) reflection is broadened at $x = 0.525$ and splits into two distinct peaks at $x > 0.525$, which could be explained by a lower symmetry with inequivalent a-axis and b-axis, i.e. an orthorhombic cell (a and $b \sim \sqrt{2}\,a_{\mathrm{tetra}}$, $c = c_{\mathrm{tetra}}$) with a sub-group *Cmm2* of $P\bar{4}2_1m$. The XRD patterns (Figure S2.1) suggested that the as-made samples $x > 0.5$ actually contained both tetragonal and orthorhombic phases and the orthorhombic phase dominates in the samples $x \geq 0.55$. The cell parameters for orthorhombic and tetragonal $La_{1+x}Ca_{1-x}Ga_3O_{7+0.5x}$, shown in Figure S2.2, become constant after $x = 0.65$ which suggests that the solid solution terminates at $x = 0.65$. The XRD pattern of the as-made $La_{1.64}Ca_{0.36}Ga_3O_{7.32}$ showed a pure orthorhombic phase with no apparent residual tetragonal phase in the sample. EDS (energy-dispersive spectroscopy) elemental analysis in the TEM investigation showed a homogenous cation ratio $La_{1.62}Ca_{0.36}Ga_3$ for $La_{1.64}Ca_{0.36}Ga_3O_{7.32}$ against $La_{1.01}Ca_{0.93}Ga_3$ from the EDS analysis for the parent material $LaCaGa_3O_7$.

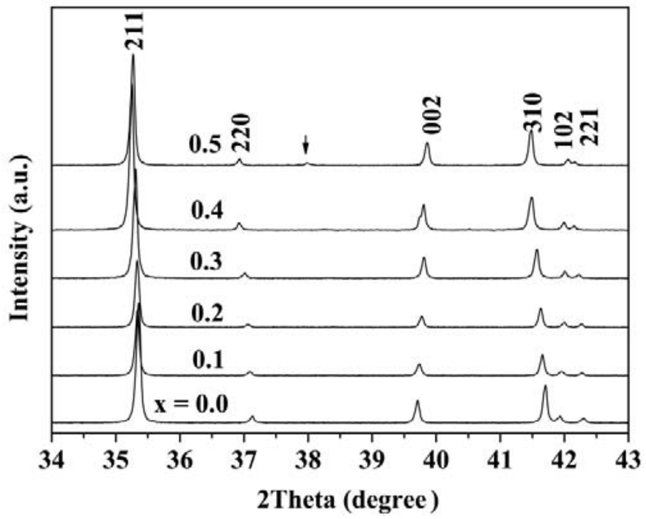

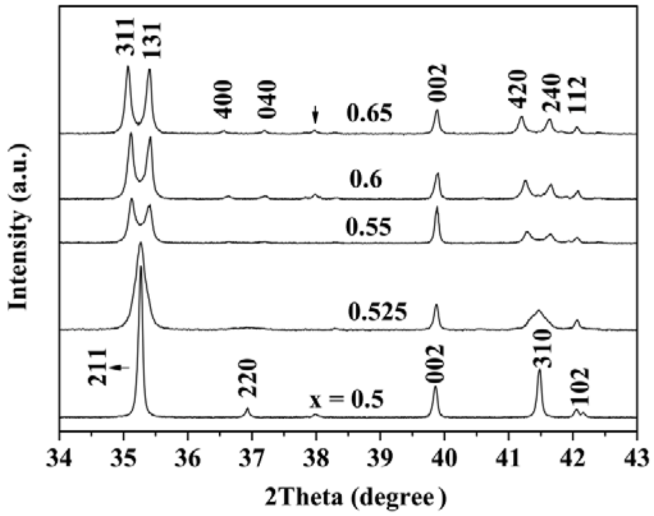

Figure S2.1. XRD patterns of as-made $La_{1+x}Ca_{1-x}Ga_3O_{7+0.5x}$. (a) $x \leq 0.5$ adopting the tetragonal cell and (b) $x \geq 0.55$ indexed with the orthorhombic cell. The arrows mark reflections from the second phase Ca-doped $LaGaO_3$.

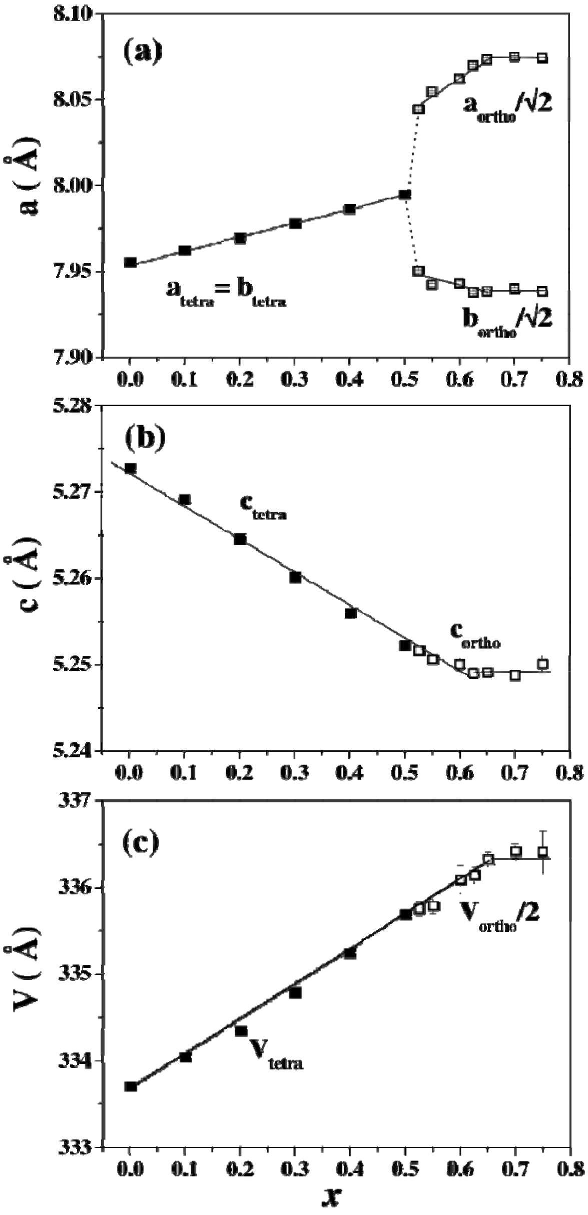

Figure S2.2. Cell parameters versus compositions for the $La_{1+x}Ca_{1-x}Ga_3O_{7+0.5x}$ solid solution. The orthorhombic cell parameters a_{ortho} and b_{ortho} were divided by $\sqrt{2}$ and volume by 2 for comparison with tetragonal cell. The tetragonal and orthorhombic cells are represented by using filled and open squares, respectively.

3. SAED and HRTEM analysis

SAED results showed that, although the main diffraction features were consistent with the crystal structure proposed by XRD (space group *Cmm2*, a =11.42 Å, b = 11.23 Å and c = 5.25 Å), extra weak reflections were observed in the diffraction patterns along certain crystal axes. These extra spots can be indexed simply by doubling the c axis of the proposed unit cell. The reciprocal lattice reconstruction indicates a c-axis doubled body-centered supercell based on the above *Cmm2* cell with no other systematic absences observable, suggesting possible space groups *Imm2*, *Im11*, and even in triclinic cell *I1*. Figure S3.2 shows the simulated SAED patterns for $La_{1.64}Ca_{0.36}Ga_3O_{7.32}$ according the the triclinic cell (Space group of *P1*, a = 9.5735 Å, b = 9.5754 Å, c = 9.5691 Å, α = 106.78°, β = 108.17°, γ = 113.51°, its relationship to the *I1* cell is shown in Figure S3.1)

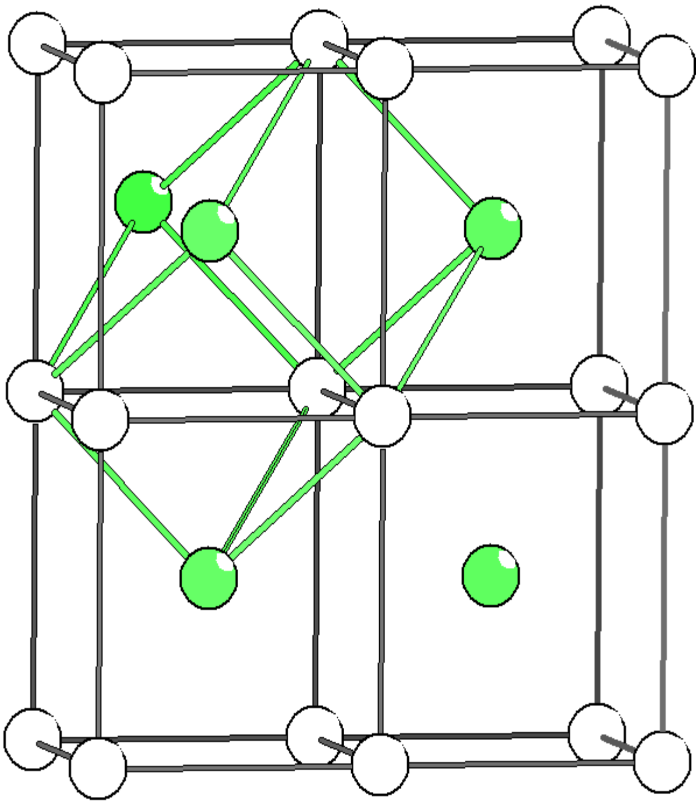

Figure S3.1 The relationship between body-centred (*I*) and primitive (*P*) unit cells, where the *I*-cell edges are black, *P*-cell edges green, and the green spheres are the centres of *I*-cell.

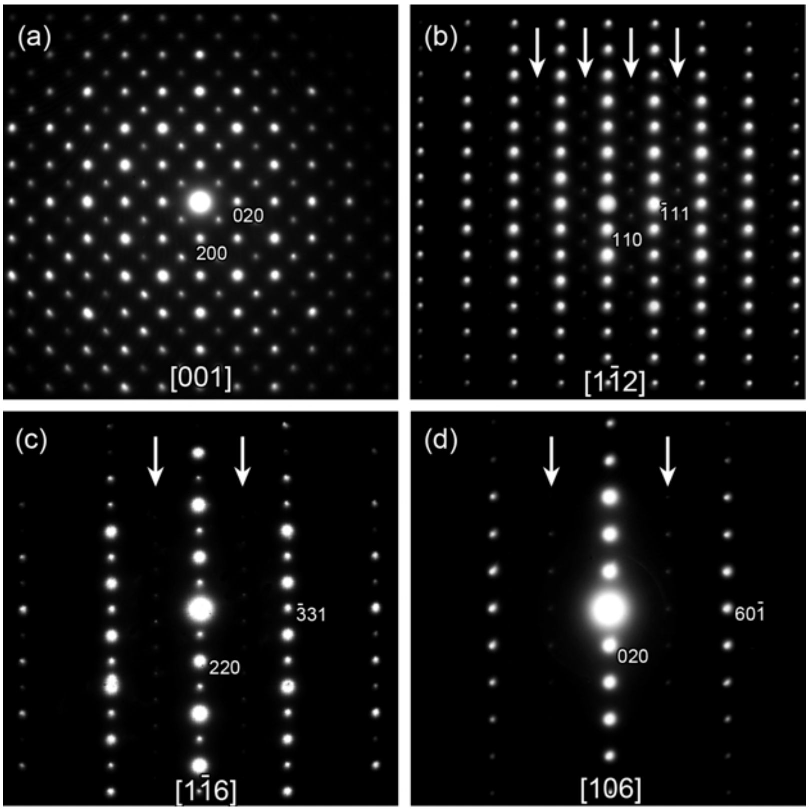

Figure S3.2 SAED patterns for $La_{1.64}Ca_{0.36}Ga_3O_{7.32}$ along the (a) [001] axis where no extra reflections are observed, and the (b) [1 $\bar{1}$ 2] (c) [1 $\bar{1}$ 6] and (d) [106] axes where superlattice reflections (as marked by white arrows), which can be indexed as $(hk\frac{2n+1}{2})$, indicate the c-doubling. The axes and reflections are indexed according to the*P1* cell.

HRTEM was applied to check possible microstructural crystal defects, such as twinning, in order to investigate any relationship with the weak diffraction features. Figure S3.3 is the HRTEM image along [112] (left) and [121] (right) simulated in the triclinic c-doubled supercell in *P1* with the final refined structural model. The images simulated from different directions are quite similar, and it is thus clear that the extra weak reflections in SAED are intrinsic features, instead of arising from microscopic defects, and responsible for the doubled *c*-axis.

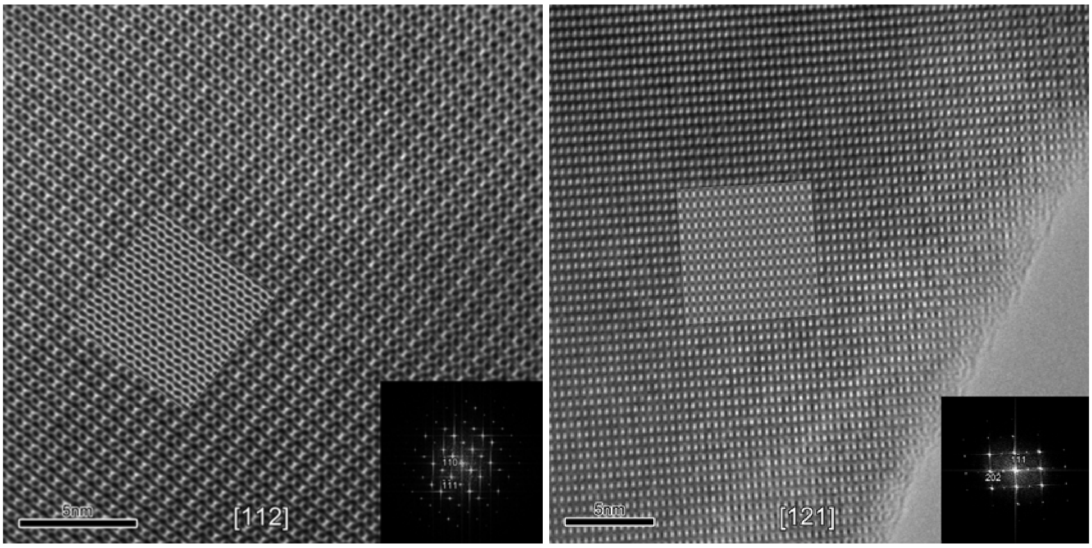

Figure S3.3 HRTEM of $La_{1.64}Ca_{0.36}Ga_3O_{7.32}$ along [112](left) and [121](right) in *P1* cell. Inset is the simulated image from the final refined model along this direction.

4. Phase transition (Variable temperature NPD)

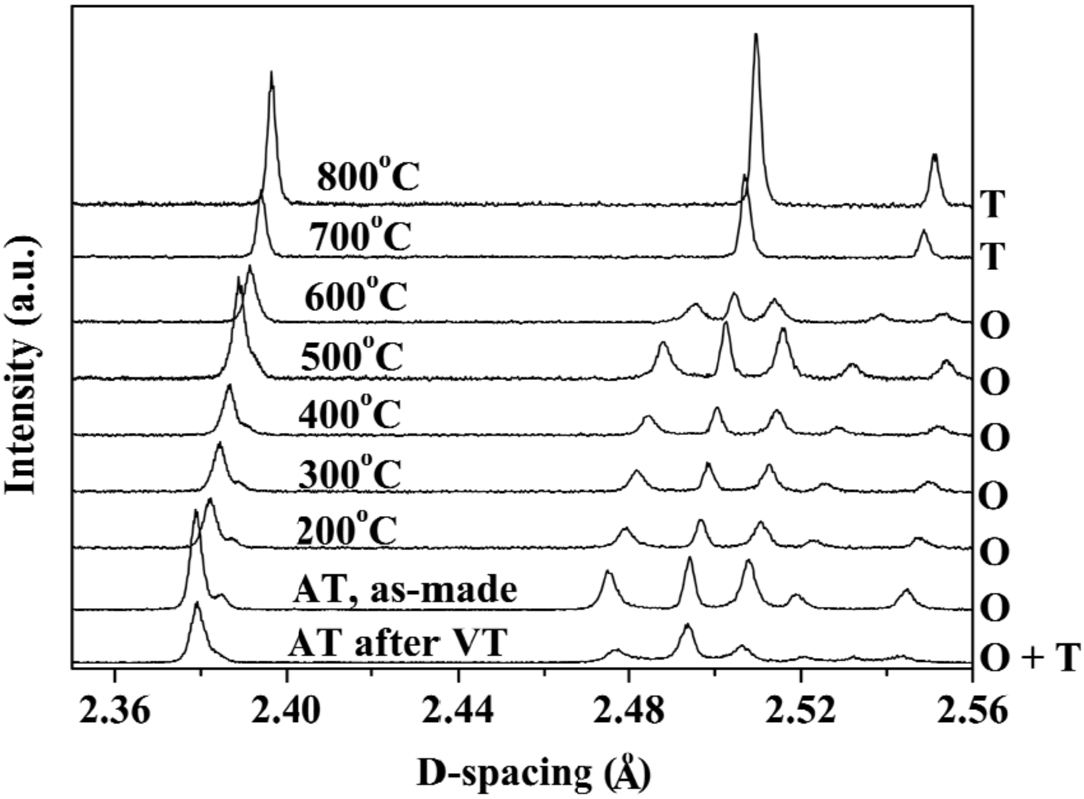

Figure S4.1 Variable temperature (VT) time-of-flight neutron diffraction (TOF ND) patterns for $La_{1.64}Ca_{0.36}Ga_3O_{7.32}$ upon heating show a phase transition between 600 and 700 °C. The phases present at each temperature are marked as orthorhombic (O) and tetragonal (T) phases. The O and T phases coexist as shown in the ambient temperature (AT) data after VT experiment, compared with the AT data for as-made sample (O phase only).

5. Structure refinement for $La_{1.64}Ca_{0.36}Ga_3O_{7.32}$ in average models

High resolution neutron HRPD (TOF) data from both backscattering (NPD168) and 90 degree (NPD90) detectors were used for crystal structure refinement in GSAS. Refinements were carried out in the following sequence (Figure S5.1), in order to explore the possible space groups suggested by SAED.

Tetra. ($P\bar{4}21m$)

↓

Ortho. (Cmm2) (a,b ~ $2^{1/2}a_{tetra}$; c ~ c_{tetra})
a, b, c = 11.416, 11.226, 5.248 Å

↓

Ortho. (Imm2) (a, b ~ $2^{1/2}a_{tetra}$; c ~ 2*c_{tetra})
a, b, c = 11.416,11.228,10.497Å

↓

Mono. (Im11) (a, b, c ~ a_{ortho}, b_{ortho}, c_{ortho})
a, b, c = 11.416,11.228,10.497Å
$\alpha = 90.02^0$

↓

Tri. (P1) (a, b, c ~ a_{ortho}, b_{ortho}, c_{ortho}-I1)
a, b, c = 9.573, 9.576, 9.569
α, β, γ = 106.78, 108.17, 113.51^0

Figure S5.1 Schematic diagram for structure refinement

The general parameters, including histogram scale factors, background coefficients, unit cell parameters, zero error and peak shape coefficients, were varied for all refinements. Framework atoms of the starting model were derived from tetragonal melilite phase and the extra oxygen sites were

established using Fourier synthesis techniques and refined in accordance with nominal composition and charge balance. The atomic positional and displacement parameters of mixed site atoms were constrained to be the same. In the split-site model, the atomic displacement parameters (ADPs) for split sites were refined isotropically for convergence, and the related values for bulk and defect structure were simultaneously refined to be the same value. The final refined results were shown in Table S5.1.

Table S5.1 The cell parameters, refined variables (RV), and fitted parameters from the final refinement in S.G. *Cmm2*, *Imm2*, *Im11*, *P1* (average model (*P1*-average) and split model (*P1*-split)) for comparing.

**S.G.**	***Cmm2***	***Imm2***	***Im11***	***P1*-average**	***P1*-split**
**a (Å)**	11.41253(6)	11.41582(3)	11.41608(3)	9.57355(3)	9.57230(4)
**b (Å)**	11.22407(6)	11.22801(3)	11.22797(3)	9.57482(3)	9.57283(4)
**c (Å)**	5.24802(9)	10.49733(3)	10.49736(2)	9.56915(3)	9.56967(4)
**α (°)**	90	90	90.023(1)	106.781(1)	106.779(1)
**β (°)**	90	90	90	108.173(1)	108.176(1)
**γ (°)**	90	90	90	113.510(1)	113.501(1)
**V ($Å^3$)**	672.24(2)	1345.516(6)	1345.547(5)	672.846(4)	672.726(4)
**RV**	122	201	320	536	507
**R_{wp}/R_p%**	4.81/5.47	4.12/5.37	3.37/4.96	3.21/4.60	3.30/4.42
**χ^2**	9.31	6.84	4.69	4.28	4.81

5.1 Orthorhombic cell, *Cmm2* space group

The Rietveld refinement plots of data in the *Cmm2* cell (see Section 3 and Figure S5.1) are shown in Figure S5.2 (R_{wp}/R_p = 4.81/5.47%, χ^2 = 9.31). As can be seen in Figure S5.2, the fit is poor, especially in the high d-spacing range (~ 2.4 Å). No significant improvement on the fitting was achieved by adding a tetragonal phase in the refinement, indicating that there is no minor quenched tetragonal phase. Excess weak reflections could be examined as shown in the inset of Figure S5.2a, which requires a larger supercell. Thus, the following refinements were carried out considering the c-axis doubled supercell as discussed above (Section 3 and Figure S5.1).

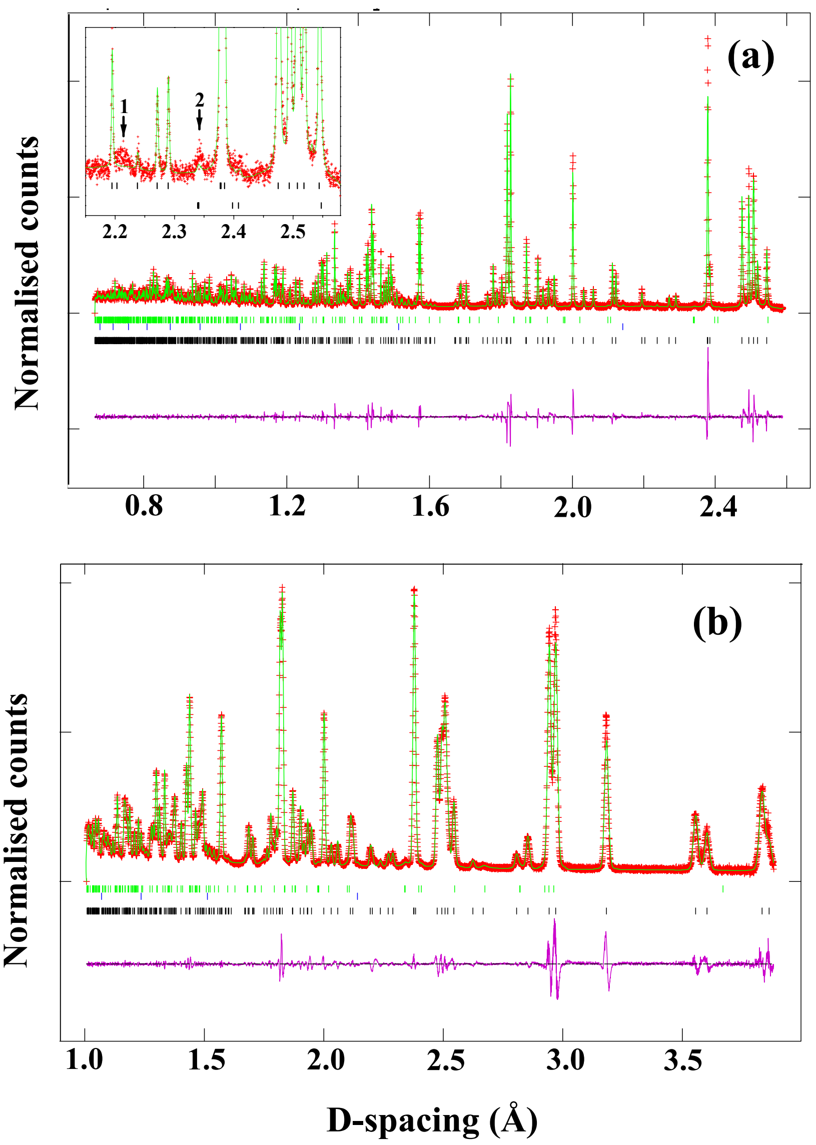

Figure S5.2 Rietveld refinement of RT HRPD ND data in S.G. *Cmm2* from (a) backscattering (b) 90 degree banks. Three rows of vertical tick mark the reflections from $La_{1.64}Ca_{0.36}Ga_3O_{7.32}$, the vanadium container, and Ga_2O_3 from bottom to top, respectively. The enlarged inset of (a) between 2.15-2.56Å of to show two set of weak reflections, which cannot be accounted for by the *Cmm2* cell, requiring a *I* c-doubling cell. The impurity Ga_2O_3 has contribution to the weak reflections around 2.35 Å marked as 2.

5.2 Orthorhombic cell, *Imm2* space group

The followed Rietveld refinement in the c-doubled supercell in *Imm2* still gave poor fitting (Figure S5.3; R_{wp}/R_p = 4.12/5.37%, χ^2 = 6.84). The fit cannot be improved by switching to the Stephen's anisotropic broadening function[2] or by adding the tetragonal phase into the refinement, which suggested the investigation of lower symmetry for $La_{1.64}Ca_{0.36}Ga_3O_{7.32}$.

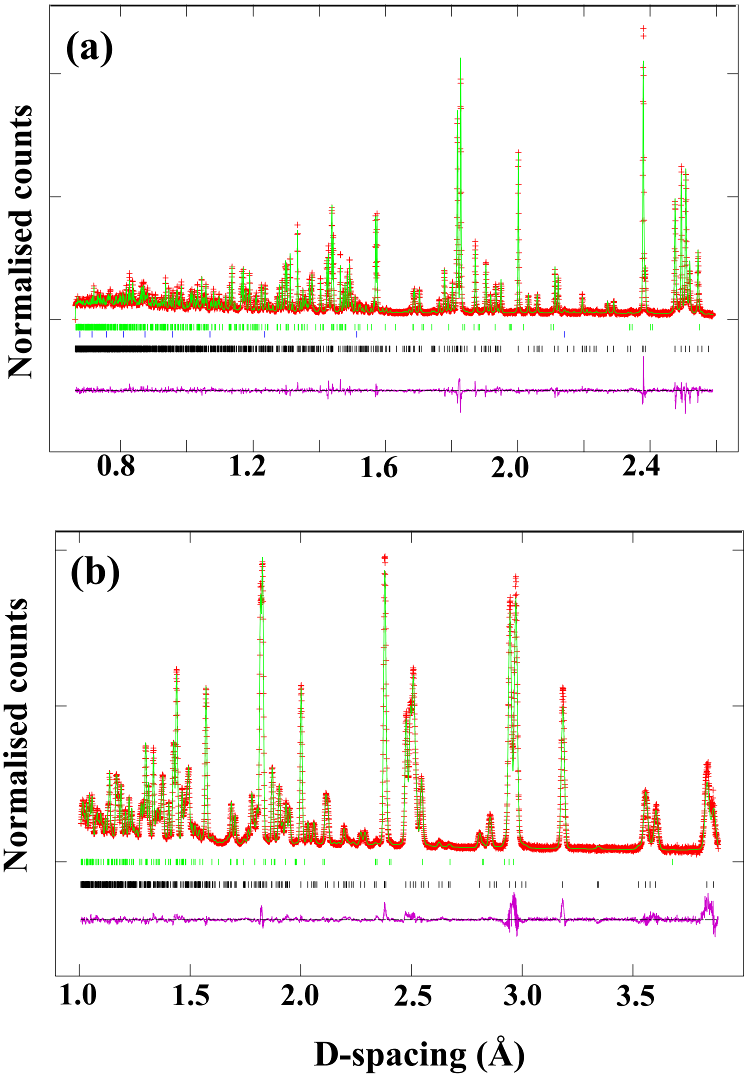

Figure S5.3 Rietveld refinement of RT HRPD ND data in S.G. *Imm2* from (a) backscattering (b) 90 degree banks. Three rows of vertical tick mark the reflections from $La_{1.64}Ca_{0.36}Ga_3O_{7.32}$, the vanadium container, and Ga_2O_3 from bottom to top, respectively

5.3 Monoclinic cell, *Im11* space group

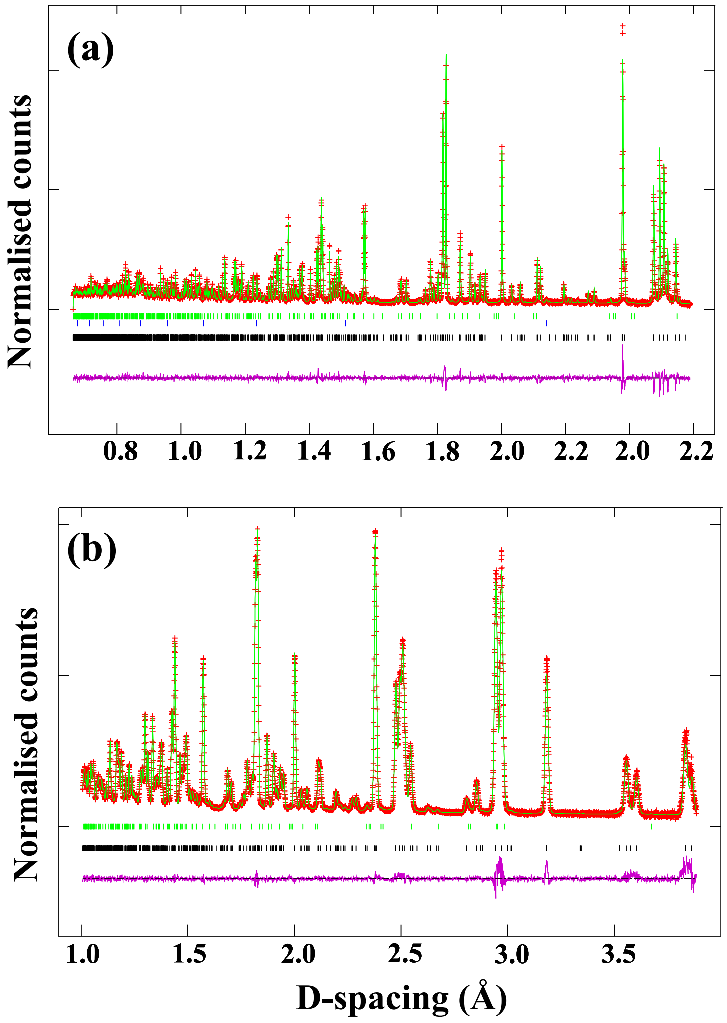

Figure S5.4 Rietveld refinement of RT HRPD ND data based on the average model in S.G. *Im11* from (a) backscattering (b) 90 degree banks. Three rows of vertical tick mark the reflections from $La_{1.64}Ca_{0.36}Ga_3O_{7.32}$, the vanadium container, and Ga_2O_3 from bottom to top, respectively.

The Rietveld refinement in the lower symmetry monoclinic space group *Im11* is significantly improved as shown in Figure S5.4 compared with Figure S5.3 and converged to $R_{wp}/R_p = 3.37/4.96\%$, and $\chi^2 = 4.69$. Three distinct interstitial sites were revealed and the total population was constrained to be 2.56 for charge balance as listed in Table S5.2 and Figure S5.5. However, a slight misfit in the high d-spacing range (~2.0 Å) could be observed (Figure S5.4), implying lower symmetry and/or more

ordered extra oxide distribution. Therefore, further refinement was performed in a triclinic cell (*P1*) by removing the mirror symmetry element in Figure S5.5.

Table S5.2 Refined structural parameters for $La_{1.64}Ca_{0.36}Ga_3O_{7.32}$ at room temperature for the average structure in monoclinic *Im11*

Atom	Site	x	y	z	Occupancy
La/Ca1	4*b*	0.3367(1)	0.4926(2)	0.7498(1)	0.780(1)/0.220(1)[a]
La/Ca2	4*b*	0.3371(1)	0.4904 (3)	0.2496(1)	0.984(1)/0.016(1)[a]
La/Ca3a	2*a*	0.5	0.8309(2)	0.7538(3)	0.915(1)/0.085(1)[a]
La/Ca3b	2*a*	0.5	0.1529(2)	0.7589(2)	0.620(1)/0.380(1)[a]
La/Ca4a	2*a*	0.5	0.8250(2)	0.2621(2)	0.651(1)/0.349(1)[a]
La/Ca4b	2*a*	0.5	0.1534(2)	0.2464(2)	0.846(1)/0.154(1)[a]
Ga1a	4*b*	0.2520(3)	0.7432(3)	0.4961(2)	1
Ga1b	4*b*	0.7491(3)	0.2423(3)	0.4969(2)	1
Ga2a	4*b*	0.1351(1)	0.4858(3)	0.5165(1)	1
Ga2b	4*b*	0.1371(1)	0.4851(2)	0.0170(1)	1
Ga3a1	2*a*	0.5	0.6368(2)	0.9818(2)	1
Ga3a2	2*a*	0.5	0.3360(2)	0.9805(2)	1
Ga3b1	2*a*	0.5	0.6378(2)	0.4867(2)	1
Ga3b2	2*a*	0.5	0.3438(2)	0.4823(2)	1
O1	2*a*	0.5	0.4871(4)	0.9088(2)	1
O2	2*a*	0.5	0.4926(4)	0.4044(2)	1
O3a	2*a*	0.5	0.0236(2)	0.5978(3)	0.704(8)[b]
O3b	2*a*	0.5	0.9884(11)	0.6074(6)	0.296(8)[b]
O4a	2*a*	0.5	-0.0047(11)	0.0952(5)	0.444(6)[c]
O4b	2*a*	0.5	0.9377(4)	0.0815(6)	0.556(6)[c]
O5	4*b*	0.1376(1)	0.4923(4)	0.8454(1)	1
O6	4*b*	0.1376(1)	0.4931(3)	0.3441(1)	1
O7a	2*a*	0.5	0.6286(2)	0.6580(3)	1
O7b	2*a*	0.5	0.3502(2)	0.6548(2)	1
O8a	2*a*	0.5	0.6238(3)	0.1540 (3)	1
O8b	2*a*	0.5	0.3538(2)	0.1501(2)	1
O9a	4*b*	0.2384(2)	0.5998(2)	0.0802(2)	1
O9b	4*b*	0.7897(2)	0.36108(2)	0.1053(2)	1
O10a	4*b*	0.2151(2)	0.6140(2)	0.6006(2)	1
O10b	4*b*	0.7895(2)	0.3630(2)	0.6020(2)	1
O11a	4*b*	0.8739(2)	0.2075(2)	0.3936(2)	1
O11b	4*b*	0.1383(2)	0.8017(2)	0.3938(2)	1
O12a	4*b*	0.8722(2)	0.1959(2)	0.8932(2)	1
O12b	4*b*	0.1235(1)	0.7790(1)	0.8923(2)	1
O13	4*b*	0.490(2)	0.1459(4)	0.0152(7)	0.155(2)[d]
O14a	2*a*	0.5	0.8271(3)	0.4961(4)	0.673(1)[d]
O14b	2*a*	0.5	0.1550(6)	0.484(3)	0.294(2)[d]

Space group: *Im11*; $Z = 8$; $a = 11.4161(1)$ Å, $b = 11.2280(1)$ Å, $c = 10.4974(1)$, $\alpha = 90.02(1)^0$, $V = 1345.55(1)$ Å^3; R_{wp}/R_p(NPD168) = 6.41/6.30%; R_{wp}/R_p(NPD90) = 2.90/3.60%; R_{wp}/R_p (Total) = 3.37/4.97%; and $\chi^2 = 4.72$.

[a] Occupancies of mixed La/Ca site were constrained to be unity and the total La/Ca = 6.56/1.44

[b,c] Occupancy (Occ.) sum of O3a and O3b was constrained to be unity (Occ.O3a + Occ. O3b = 1), as for O4a and O4b (Occ.O4a + Occ. O4b = 1).

[d] The interstitial oxide content was constrained according to the charge-balanced nominal composition at 2.56 in the unit cell.

**Atom**	**U_{11}(Å^2)**	**U_{22}(Å^2)**	**U_{33}(Å^2)**	**U_{12}(Å^2)**	**U_{13}(Å^2)**	**U_{23}(Å^2)**
La/Ca1[a]	0.0055(5)	0.0058(4)	0.0060(4)	0.001(2)	0.0001(6)	0.0015(13)
La/Ca2[a]	0.0207(5)	0.0295(5)	0.0369(6)	-0.008(2)	-0.0039(7)	0.001(2)
La/Ca3a[a]	0.053(2)	0.053(1)	0.056(1)	0	0	-0.002(2)
La/Ca3b[a]	0.0065(10)	0.0060(9)	0.0066(10)	0	0	0.001(1)
La/Ca4a[a]	0.0134(11)	0.0132(10)	0.0205(11)	0	0	-0.004(1)
La/Ca4b[a]	0.0256(11)	0.0122(10)	0.0348(10)	0	0	0.005(1)
Ga1a	0.0092(5)	0.0093(5)	0.0103(4)	-0.0004(5)	0.003(2)	0.004(2)
Ga1b	0.0099(5)	0.0090(5)	0.0175(5)	0.0034(5)	0.007(2)	-0.005(2)
Ga2a	0.0237(6)	0.0361(7)	0.0260(7)	0.001(2)	-0.002(1)	-0.014(2)
Ga2b	0.0056(5)	0.0136(5)	0.0079(3)	0.001(1)	-0.0020(5)	-0.007(1)
Ga3a1	0.0069(9)	0.0114(10)	0.0118(10)	0	0	0.0072(11)
Ga3a2	0.0084(10)	0.0027(9)	0.0059(9)	0	0	-0.0039(9)
Ga3b1	0.087(11)	0.0228(1)	0.0225(12)	0	0	-0.003 (1)
Ga3b2	0.0149(10)	0.0206(11)	0.0141(11)	0	0	-0.004(1)
O1	0.034(1)	0.034(1)	0.024(1)	0	0	-0.016(4)
O2	0.0113(10)	0.0146(10)	0.001(1)	0	0	0.001(3)
O3a/O3b[a]	0.0021(11)	0.026(2)	0.009(1)	0	0	0.009(2)
O4a/O4b[a]	0.048(2)	0.071(4)	0.037(2)	0	0	-0.010(3)
O5	0.0243(9)	0.0290(9)	0.0255(8)	-0.006(3)	-0.0030(9)	0.001(2)
O6	0.0052(7)	0.0055(6)	0.0052(6)	0.001(1)	0.0003(7)	0.002(2)
O7a	0.019(1)	0.013(11)	0.0016(11)	0	0	0.001(1)
O7b	0.0018(11)	0.0019(1)	0.0018(1)	0	0	0.0002(5)
O8a	0.052(2)	0.034(2)	0.036(2)	0	0	-0.015(2)
O8b	0.0016(11)	0.0018(11)	0.0021(11)	0	0	0.0002(1)
O9a	0.0222(11)	0.050(1)	0.043(1)	0.0012(11)	-0.011(1)	0.002(1)
O9b	0.0245(9)	0.0103(8)	0.0124(9)	-0.0029(8)	0.0049(10)	-0.002(1)
O10a	0.0280(11)	0.0279(11)	0.0201(11)	-0.0066(9)	-0.009(1)	0.0154(10)
O10b	0.0364(11)	0.0214(10)	0.0203(11)	-0.009(1)	0.006(1)	0.001(1)
O11a	0.045(1)	0.057(1)	0.027(1)	0.014(1)	-0.004(1)	0.006(1)
O11b	0.0171(10)	0.0354(11)	0.0114(9)	0.0014(9)	-0.004(1)	-0.003(1)
O12a	0.0290(11)	0.047(1)	0.0249(11)	0.0114(10)	-0.004(1)	0.007(1)
O12b	0.0037(8)	0.0056(8)	0.0045(7)	0.0015(7)	-0.003(1)	-0.001(1)
O13	0.006(5)	0.008(4)	0.008(4)	0.004(11)	-0.005(1)	-0.005(4)

O14a	0.018(2)	0.038(2)	0.018(2)	0	0	-0.003(3)
O14b	0.024(9)	0.47(4)	0.55(5)	0	0	-0.22(4)

[a] ADPs of mixed La/Ca and disordered O3a/O3b, O4a/O4b pairs were constrained to be the same value.

Table S5.3 Selected inter-atomic distances and BVS calculations for $La_{1.64}Ca_{0.36}Ga_3O_{7.32}$ at room temperature from the average model in monoclinic cell (S.G. *Im11*)

**Bond**		**Length(Å)**		**Bond**		**Length(Å)**	
La/Ca1	O5	2.485(2)	(x1)	Ga1a	O11b	1.807(4)	(x1)
	O10a	2.498(3)	(x1)		O9b	1.803(3)	(x1)
	O1	2.507(2)	(x1)		O12a	1.825(4)	(x1)
	O10b	2.569(3)	(x1)		O10a	1.867(3)	(x1)
	O7a	2.595(3)	(x1)	BVS for Ga1a		3.10	
	O11b	2.639(3)	(x1)				
	O7b	2.650(2)	(x1)	Ga1b	O10b	1.808(4)	(x1)
	O11a	2.877(3)	(x1)		O9a	1.827(4)	(x1)
BVS for La/Ca1		2.62/1.50			O11a	1.833(4)	(x1)
					O12b	1.853(4)	(x1)
				BVS for Ga1b			
La/Ca2	O9a	2.437(3)	(x1)			3.05	
	O2	2.470(2)	(x1)	Ga2a	O4a	1.753(3)	(x1)
	O6	2.484(2)	(x1)		O4b	1.771(3)	(x1)
	O9b	2.550(3)	(x1)		O6	1.813(2)	(x1)
	O8a	2.590(3)	(x1)		O10b	1.857(3)	(x1)
	O8b	2.627(3)	(x1)		O10a	1.919(3)	(x1)
	O12a	2.785(4)	(x1)	BVS for Ga2a		3.02	
	O12b	2.842(3)	(x1)				
BVS for La/Ca2		2.70/1.55		Ga2b	O5	1.804(2)	(x1)
					O3a	1.832(2)	(x1)
La/Ca3a	O3b	2.343(11)	(x1)		O3b	1.831(3)	(x1)
	O11a	2.479(3)	(x2)		O9b	1.870(3)	(x1)
	O7a	2.483(4)	(x1)		O9a	1.854(3)	(x1)
	O6	2.585(3)	(x2)	BVS for Ga2b		2.98	
	O14a	2.705(5)	(x1)				
	O3a	2.714(4)	(x1)	Ga3a1	O8a	1.814(4)	(x1)
	O9b	2.882(2)	(x2)		O1	1.845(5)	(x1)
BVS for La/Ca3a		2.84/1.63			O11a	1.8874(3)	(x2)
				BVS for Ga3a1		2.84	
La/Ca3b	O3a	2.228(4)	(x1)	Ga3a2	O8b	1.791(3)	(x1)
	O3b	2.437(10)	(x1)		O1	1.853(5)	(x1)
	O7b	2.471(3)	(x1)		O11b	1.863(2)	(x2)
	O6	2.547(3)	(x2)		O13	2.169(5)	(x2)
	O11b	2.701(3)	(x2)	BVS for Ga3a2		3.57	
	O13	2.694(8)	(x2)				
	O14b	2.89(3)	(x1)	Ga3b1	O7a	1.802(3)	(x1)

BVS for La/Ca3b	3.03/1.74		
La/Ca4a	O4b	2.279(6)	(x1)
	O14a	2.457(6)	(x1)
	O12a	2.475(3)	(x2)
	O8a	2.528(4)	(x1)
	O4a	2.594(10)	(x1)
	O5	2.600(4)	(x2)
	O10b	2.963(2)	(x2)
BVS for La/Ca4a	3.15/1.81		
La/Ca4b	O4a	2.381(10)	(x1)
	O13	2.431(8)	(x2)
	O14b	2.49(3)	(x1)
	O8b	2.468(3)	(x1)
	O12b	2.515(3)	(x2)
	O5	2.611(4)	(x2)
	O10a	2.927(2)	(x2)
	O4b	2.976(6)	(x1)
BVS for La/Ca4b	3.35/1.92		
	O2	1.845(5)	(x1)
	O12a	1.875(3)	(x2)
	O14a	2.127(4)	(x1)
BVS for Ga3b1	3.25		
Ga3b2	O7b	1.813(3)	(x1)
	O12b	1.846(2)	(x2)
	O2	1.860(5)	(x1)
	O14b	2.120(7)	(x1)
BVS for Ga3b2	3.31		
O3a	O3b	0.408(12)	(x1)
	O14b	1.90(2)	(x1)
	O14a	2.452(5)	(x1)
O3b	O14a	2.155(12)	(x1)
	O14b	2.28(2)	(x1)
O4a	O4b	0.663(13)	(x1)
	O13	1.891(12)	(x2)
O4b	O13	2.442(7)	(x2)

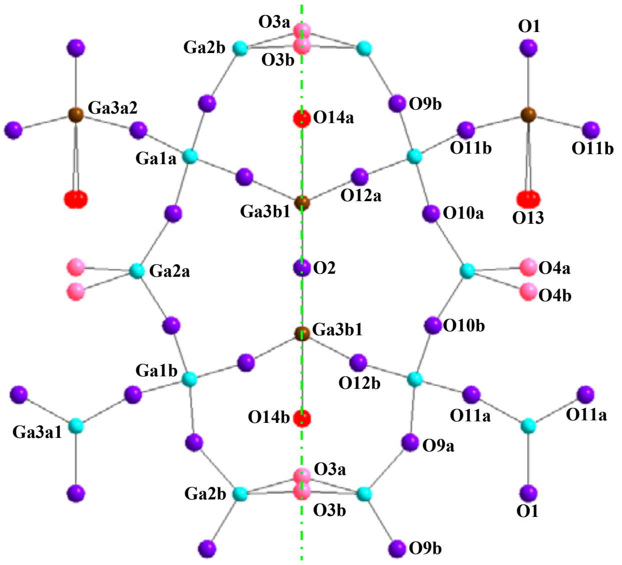

Figure S5.5 Projection view of the crystal structure along the c-axis in *Im11* symmetry. The dashed line shows the ‘mirror plane’. The A-site cations and oxygens overlapped with galliums were omitted for clarity.

Figure S5.5 shows a view of the crystal structure along the c-axis. In the crystal structure, $Ga1aO_4$ and $Ga1bO_4$ tetrahedra are 4-connected with no terminal oxygens, the other GaO_4 polyhedra are 3-

connected with one non-bridging oxygen. Two interstitial sites O14a and O14b are located on the "mirror" plane, O13 is disordered on either side of this plane. As in to $La_{1.54}Sr_{0.46}Ga_3O_{7.25}$, all the interstitial oxides are closest to tetrahedra with terminal oxygen, namely $Ga3a2O_4$, $Ga3b1O_4$, and $Ga3b2O_4$. Ga2a and Ga2b are also 3-connected with disordered bridging oxygen pairs of O4a/O4b and O3a/O3b, respectively. There is no interstitial oxide connected to Ga2a and Ga2b, leaving the candidate interstitial sites empty inside the channels neighbouring the occupied interstitials.

5.4 Triclinic cell with space group of *P1*

The optimized refinement in triclinic *P1* (R_{wp}/R_p = 3.21/4.60%, and χ^2 = 4.28, Figure S5.6) exhibited four distinct interstitial sites with total population of 1.28 as listed in Table S5.4. Compared with the crystal structure in monoclinic symmetry *Im11* (Section 5.2), all the interstitial oxides are incorporated into GaO_4 tetrahedra with terminal oxygens. Four of the A-sites show cation ordering and are occupied by La only - the occupancy of La was set to be one if it refined to be greater than 0.98. The other four A-sites are disordered and the occupancies were refined to yield a total La/Ca ratio of 6.54/1.44 (Table S5.4). Refinement of a random A-site distribution (La/Ca = 0.82/0.18 for each site) led to a poorer fit (R_{wp}/R_p = 3.49/4.84%, χ^2 = 4.96), which recovered to the previous A-site cation order after the occupancies were refined.

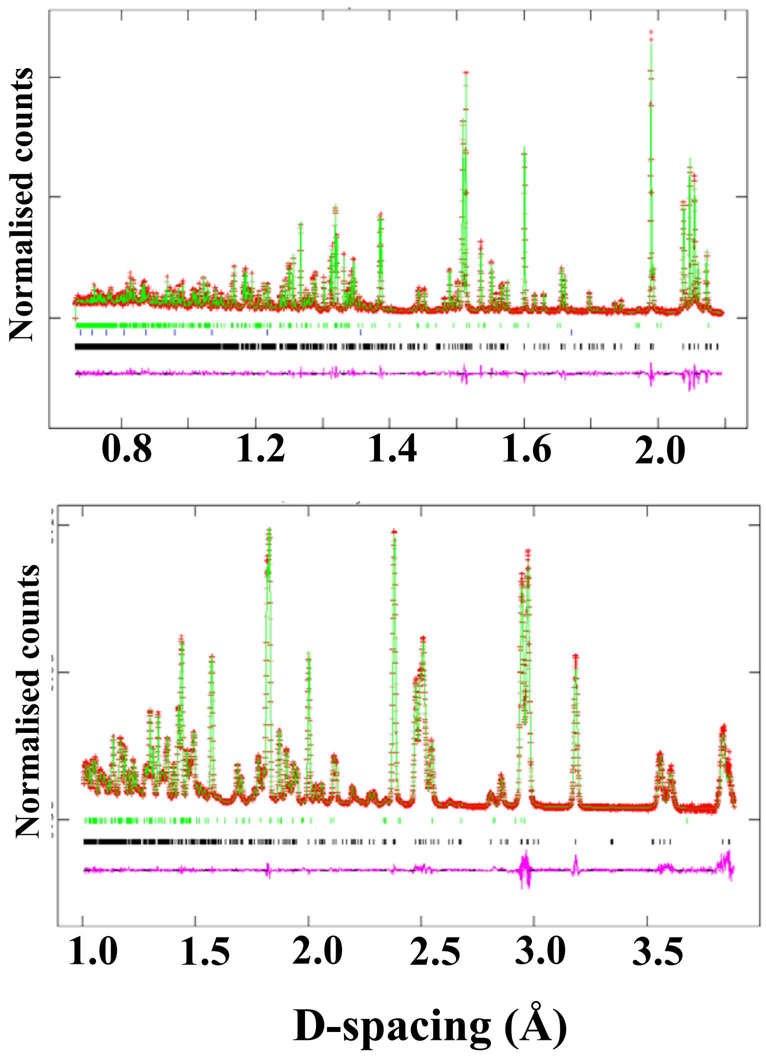

Figure S5.6 Rietveld refinement of RT HRPD ND data for the average model in *P1* from (a) backscattering (b) 90 degree bank. Three rows of vertical ticks mark the reflections from $La_{1.64}Ca_{0.36}Ga_3O_{7.32}$, the vanadium container, and Ga_2O_3 from bottom to top, respectively

Table S5.4 Refined structural parameters for $La_{1.64}Ca_{0.36}Ga_3O_{7.32}$ at room temperature for the average structure model in *P1*.

Atom	Site	*x*	*y*	*z*	Occupancy
La1a	1a	0.2395(2)	0.0877(2)	0.8281(2)	1
La/Ca1b	1a	0.2390(2)	0.4077(3)	0.1503(2)	0.814(1)/0.186(1)[a]
La2a	1a	0.7411(3)	0.5894(3)	0.8235(3)	1
La2b	1a	0.7325(2)	0.9107(3)	0.1506(2)	1

La3a	1a	0.5777(3)	0.2456(4)	0.3282(3)	1
La/Ca3b	1a	0.9118(3)	0.2640(3)	0.6550(3)	0.312(1)/0.688(1)[a]
La/Ca4a	1a	0.0860(2)	0.7608(3)	0.3237(3)	0.623(1)/0.377(1)[a]
La/Ca4b	1a	0.4022(3)	0.7509(2)	0.6536(2)	0.811(1)/0.189(1)[a]
Ga1a	1a	0.2323(4)	0.7434(4)	0.9860(4)	1
Ga1b	1a	0.2346(4)	0.2472(5)	0.4888(5)	1
Ga1c	1a	0.7319(5)	0.2385(4)	0.9810(4)	1
Ga1d	1a	0.7409(4)	0.7462(4)	0.4935(4)	1
Ga2a	1a	0.0023(2)	0.6555(3)	0.6233(2)	1
Ga2b	1a	0.0086(3)	0.3778(3)	0.3556(3)	1
Ga2c	1a	0.5084(2)	0.1534(2)	0.6255(2)	1
Ga2d	1a	0.5042(2)	0.8835(3)	0.3487(2)	1
Ga3a1	1a	0.6189(2)	0.4804(2)	0.1354(2)	1
Ga3a2	1a	0.3202(2)	0.4781(3)	0.8359(3)	1
Ga3b1	1a	0.1240(3)	0.9855(3)	0.1354(3)	1
Ga3b2	1a	0.8247(2)	0.9825(3)	0.8407(3)	1
O1	1a	0.3942(4)	0.4074(4)	0.9853(4)	1
O2	1a	0.8924(3)	0.9002(3)	0.9812(3)	1
O3a	1a	0.6267(4)	0.0856(4)	0.5294(4)	0.618(5)[b]
O3b	1a	0.5758(6)	0.0985(6)	0.4753(6)	0.382(5)[b]
O4a	1a	0.0958(9)	0.5927(8)	0.5029(8)	0.272(12)[c]
O4b	1a	0.0530(4)	0.5911(5)	0.4562(4)	0.728(12)[c]
O5a	1a	0.3367(3)	0.9809(3)	0.6249(3)	1
O5b	1a	0.3393(3)	0.7092(3)	0.3542(3)	1
O6a	1a	0.8382(3)	0.48417(29)	0.6314(3)	1
O6b	1a	0.8303(3)	0.2048(4)	0.3478(3)	1
O7a	1a	0.2877(3)	0.1566(3)	0.1268(3)	1
O7b	1a	0.0056(3)	0.1541(3)	0.8493(3)	1
O8a	1a	0.7779(3)	0.6501(3)	0.1216(3)	1
O8b	1a	0.5053(3)	0.6530(3)	0.8541(3)	1
O9a	1a	0.6767(4)	0.3178(5)	0.8344(5)	1
O9b	1a	0.6998(4)	0.8656(4)	0.3870(5)	1
O9c	1a	0.4697(4)	0.8887(4)	0.1435(4)	1
O9d	1a	0.4675(3)	0.3194(3)	0.5831(3)	1
O10a	1a	0.2110(4)	0.8137(4)	0.8281(4)	1
O10b	1a	0.2304(4)	0.4080(4)	0.4271(4)	1
O10c	1a	0.9664(4)	0.3801(4)	0.1482(4)	1
O10d	1a	0.9690(3)	0.8159(3)	0.5840(3)	1
O11a	1a	0.6277(5)	0.2858(5)	0.1047(5)	1
O11b	1a	0.5937(4)	0.5188(4)	0.3266(4)	1
O11c	1a	0.1844(3)	0.5249(3)	0.9256(3)	1
O11d	1a	0.1962(4)	0.2512(4)	0.6644(4)	1
O12a	1a	0.1043(4)	0.7740(4)	0.0825(4)	1

O12b	1a	0.0763(4)	0.0306(5)	0.3111(4)	1
O12c	1a	0.6720(3)	0.0155(31	0.9100(3)	1
O12d	1a	0.6736(3)	0.7642(4)	0.6532(3)	1
O13a	1a	0.1577(9)	0.5008(8)	0.6453(8)	0.280(1)[d]
O13b	1a	0.8211(6)	0.4903(10)	0.3172(10)	0.179(1)[d]
O14a	1a	0.3346(6)	0.9991(5)	0.3248(5)	0.582(1)[d]
O14b	1a	0.6181(15)	0.9872(12)	0.6746(11)	0.239(1)[d]

Space group: *P1*; $Z = 4$; $a = 9.5735(1)$ Å, $b = 9.5748(1)$ Å, $c = 9.5691(1)$ Å, $\alpha = 106.78(1)°$, $\beta = 108.17(1)°$, $\gamma = 113.51(1)°$, $V = 672.85(1)$ Å^3. R_{wp}/R_p%(ND168) = 6.08/5.84, R_{wp}/R_p%(ND90) = 2.77/3.33, R_{wp}/R_p%(Total)= 3.21/4.60, $\chi^2 = 4.28$.

[a] Occupancies of the mixed La/Ca site were constrained to be unity and the total La/Ca ratio 6.56/1.44

[b,c] Occupancy sum of O3a and O3b was constrained to be unity (Occ.O3a + Occ. O3b = 1); the same to O4a and O4b (Occ.O4a + Occ. O4b = 1).

[d] The interstitial oxide content was fixed according to the charge-balanced nominal composition of 1.28 in the unit cell.

**Atom**	**U_{11} (Å^2)**	**U_{22} (Å^2)**	**U_{33} (Å^2)**	**U_{12} (Å^2)**	**U_{13} (Å^2)**	**U_{23} (Å^2)**
La1a	0.0037(5)	0.0036(5)	0.0041(5)	0.0033(4)	0.0010(4)	0.0013(5)
La/Ca1b[a]	0.0087(6)	0.0098(6)	0.0054(7)	0.0081(5)	0.0019(5)	0.0037(6)
La2a	0.0399(8)	0.0441(11)	0.0471(11)	0.0291(7)	0.0209(7)	0.0078(9)
La2b	0.0263(7)	0.0344(9)	0.0289(9)	0.0188(6)	0.0172(6)	0.0112(7)
La3a	0.0441(9)	0.0463(11)	0.0474(12)	0.0279(7)	0.0207(9)	0.0172(8)
La/Ca3b[a]	0.0169(9)	0.0168(9)	0.0141(10)	0.0142(7)	0.0118(8)	0.0124(7)
La/Ca4a[a]	0.0104(7)	0.0144(9)	0.0136(9)	0.0101(6)	0.0034(7)	0.0040(7)
La/Ca4b[a]	0.0164(7)	0.0137(7)	0.0133(8)	0.0112(5)	0.0028(6)	0.0091(5)
Ga1a	0.0134(7)	0.0111(7)	0.0124(8)	0.0071(5)	0.0044(7)	0.0071(7)
Ga1b	0.0127(7)	0.0139(7)	0.0111(7)	0.0071(5)	0.0075(6)	0.0057(7)
Ga1c	0.0109(7)	0.0142(8)	0.0132(8)	0.0054(5)	0.0037(7)	0.0063(7)
Ga1d	0.0103(7)	0.0093(7)	0.0082(7)	0.0053(5)	0.0031(6)	0.0046(7)
Ga2a	0.0173(8)	0.0202(9)	0.0197(8)	0.0061(7)	0.0135(6)	0.0072(8)
Ga2b	0.0339(9)	0.0435(12)	0.040(1)	0.0119(9)	0.0267(7)	0.0117(11)
Ga2c	0.0018(6)	0.0016(6)	0.0017(6)	0.0009(5)	0.0007(5)	0.0015(6)
Ga2d	0.0146(7)	0.0212(9)	0.0163(8)	0.0051(7)	0.0142(5)	0.0070(8)
Ga3a1	0.0057(6)	0.0024(6)	0.0035(7)	0.0022(5)	0.0028(6)	0.0005(5)
Ga3a2	0.0099(7)	0.0190(9)	0.0216(9)	0.0091(6)	0.0052(7)	0.0030(7)
Ga3b1	0.0182(7)	0.0227(9)	0.0194(9)	0.0116(7)	0.0117(7)	0.0080(7)
Ga3b2	0.0145(8)	0.0206(9)	0.0231(9)	0.0090(7)	0.0105(7)	0.0057(7)
O1	0.0267(10)	0.0352(11)	0.0265(11)	0.0129(8)	0.0140(9)	0.0162(9)
O2	0.0114(8)	0.0171(9)	0.0121(9)	0.0051(7)	0.0083(7)	0.0103(7)

O3a/3b[a]	0.0031(11)	0.0032(13)	0.0030(11)	0.0001(11)	0.0025(7)	0.0001(10)
O4a/4b[a]	0.0493(15)	0.0486(18)	0.0525(17)	0.0093(14)	0.0316(11)	0.0203(14)
O5a	0.0107(10)	0.0107(11)	0.0092(10)	0.0011(9)	0.0071(8)	0.0053(9)
O5b	0.0262(12)	0.0304(13)	0.0338(14)	0.0192(10)	0.0183(9)	0.0164(11)
O6a	0.0040(9)	0.0071(10)	0.0068(10)	0.0023(8)	0.0047(7)	0.0019(8)
O6b	0.0245(12)	0.0283(14)	0.0211(12)	0.0084(11)	0.0111(9)	0.0164(11)
O7a	0.0043(8)	0.0037(9)	0.0035(9)	0.0022(7)	0.0022(8)	0.0028(7)
O7b	0.0203(12)	0.0195(11)	0.0222(11)	0.0065(10)	0.0071(10)	0.0145(8)
O8a	0.0089(9)	0.0156(11)	0.0147(11)	0.0055(9)	0.0082(9)	0.0058(8)
O8b	0.0209(11)	0.0210(12)	0.0331(14)	0.0136(9)	0.0097(11)	0.0101(10)
O9a	0.0547(16)	0.0484(19)	0.0508(20)	0.0319(12)	0.0310(14)	-0.006(2)
O9b	0.0489(17)	0.059(2)	0.078(2)	0.0346(13)	0.0332(17)	0.016(2)
O9c	0.0127(12)	0.0332(16)	0.0207(13)	0.0015(12)	0.0117(10)	0.0141(12)
O9d	0.006(1)	0.0077(10)	0.0182(12)	0.0042(8)	-0.0003(10)	0.0076(11)
O10a	0.0533(17)	0.0207(13)	0.0267(14)	0.0184(12)	0.0116(14)	0.0213(10)
O10b	0.0265(14)	0.0222(12)	0.0240(13)	0.0102(11)	0.0066(12)	0.0193(10)
O10c	0.0156(11)	0.0398(17)	0.0193(12)	0.0071(12)	0.0148(10)	0.0140(13)
O10d	0.0062(9)	0.0069(10)	0.0108(11)	0.0056(8)	0.0019(9)	0.0043(11)
O11a	0.0456(16)	0.0501(18)	0.0596(19)	0.0254(13)	0.0262(15)	0.0391(15)
O11b	0.0312(15)	0.0311(17)	0.0238(15)	0.0068(14)	0.0095(13)	0.0154(14)
O11c	0.0057(9)	0.0053(10)	0.006(1)	0.0016(8)	0.0052(9)	0.0030(9)
O11d	0.0164(12)	0.0159(14)	0.0270(13)	0.0009(11)	0.013(1)	0.0089(11)
O12a	0.0252(11)	0.0200(12)	0.0321(13)	0.0167(9)	0.0222(11)	0.0209(12)
O12b	0.0475(16)	0.0389(19)	0.0393(17)	0.0090(16)	0.0296(14)	0.0134(16)
O12c	0.0046(9)	0.0032(10)	0.0063(10)	0.0016(8)	0.0046(9)	0.0025(9)
O12d	0.0140(11)	0.0210(14)	0.0169(12)	0.0052(10)	0.0128(10)	0.0024(13)
O13a/13b[a]	0.025(3)	0.025(3)	0.026(3)	0.013(2)	0.020(2)	0.008(2)
O14a	0.035(2)	0.0115(18)	0.0145(19)	0.0155(14)	0.0122(17)	0.0114(12)
O14b	0.37(2)	0.53(5)	0.35(3)	0.16(2)	0.257(15)	-0.15(2)

[a] ADPs for La/Ca on all sites and for O3a/O3b, O4a/O4b, O13a and O13b were constrained to be the same value.

Table S5.5 Selected inter-atomic distances and BVS calculations for $La_{1.64}Ca_{0.36}Ga_3O_{7.32}$ at room temperature from the average model in *P1*.

Bond			Length (Å)	Bond			Length (Å)
La1a	-O1	(x1)	2.482(4)	Ga1a	-O12a	(x1)	1.810(6)
	-O10d	(x1)	2.505(2)		-O10a	(x1)	1.812(6)
	-O10a	(x1)	2.527(5)		-O11c	(x1)	1.815(5)
	-O5a	(x1)	2.536(4)		-O9c	(x1)	1.871(4)
	-O11d	(x1)	2.570(5)	BVS for Ga1a			3.08

	-O7a	(x1)	2.581(3)
	-O7b	(x1)	2.608(4)
BVS for La1a			2.58
La/Ca1b	-O1	(x1)	2.483(5)
	-O10c	(x1)	2.509(5)
	-O5b	(x1)	2.536(4)
	-O7a	(x1)	2.587(4)
	-O7b	(x1)	2.608(3)
	-O10b	(x1)	2.675(5)
	-O11c	(x1)	2.704(4)
	-O11b	(x1)	2.832(4)
BVS for La/Ca1b			2.50/1.44
La2a	-O2	(x1)	2.415(4)
	-O6a	(x1)	2.450(5)
	-O9a	(x1)	2.464(6)
	-O9d	(x1)	2.498(3)
	-O8a	(x1)	2.618(4)
	-O8b	(x1)	2.638(5)
	-O12d	(x1)	2.766(5)
	-O12a	(x1)	2.924(4)
BVS for La2a			2.77
La2b	-O9c	(x1)	2.417(5)
	-O6b	(x1)	2.468(4)
	-O9b	(x1)	2.510(6)
	-O2	(x1)	2.561(4)
	-O8b	(x1)	2.588(3)
	-O8a	(x1)	2.652(5)
	-O12b	(x1)	2.706(4)
	-O12c	(x1)	2.758(4)
BVS for La2b			2.76
La/Ca3a	-O3b	(x1)	2.260(8)
	-O11a	(x1)	2.433(6)
	-O7a	(x1)	2.444(4)
	-O14a	(x1)	2.552(6)
	-O6b	(x1)	2.559(5)
	-O11b	(x1)	2.563(6)
	-O13b	(x1)	2.621(9)
	-O6a	(x1)	2.640(3)

Ga1b	-O10b	(x1)	1.811(7)
	-O11d	(x1)	1.822(7)
	-O12b	(x1)	1.827(4)
	-O9d	(x1)	1.849(5)
BVS for Ga1b			3.08
Ga1c	-O12c	(x1)	1.814(5)
	-O9a	(x1)	1.816(7)
	-O11a	(x1)	1.841(7)
	-O10c	(x1)	1.875(4)
BVS for Ga1c			3.01
Ga1d	-O9b	(x1)	1.812(7)
	-O10d	(x1)	1.814(5)
	-O12d	(x1)	1.826(6)
	-O11b	(x1)	1.849(4)
BVS for Ga1d			3.09
Ga2a	-O4a	(x1)	1.778(10)
	-O10d	(x1)	1.803(5)
	-O6a	(x1)	1.805(4)
	-O4b	(x1)	1.824(5)
	-O10a	(x1)	1.886(3)
BVS for Ga2a			3.10
Ga2b	-O4a	(x1)	1.772(7)
	-O4b	(x1)	1.788(5)
	-O6b	(x1)	1.805(4)
	-O10b	(x1)	1.884(5)
	-O10c	(x1)	1.907(5)
BVS for Ga2b			2.96
Ga2c	-O3b	(x1)	1.781(7)
	-O9a	(x1)	1.794(3)
	-O5a	(x1)	1.803(3)
	-O3a	(x1)	1.855(5)
	-O9d	(x1)	1.902(5)
BVS for Ga2c			3.06
Ga2d	-O3b	(x1)	1.758(6)
	-O3a	(x1)	1.761(3)
	-O5b	(x1)	1.811(4)

	-O3a	(x1)	2.826(6)		-O9b	(x1)	1.880(5)
	-O9c	(x1)	2.903(5)		-O9c	(x1)	1.906(5)
	-O9d	(x1)	2.960(5)	BVS for Ga2d			3.01
BVS for La/Ca3a			2.88/1.66				
				Ga3a1	-O8a	(x1)	1.803(4)
La/Ca3b	-O3a	(x1)	2.205(4)		-O11a	(x1)	1.841(6)
	-O7b	(x1)	2.492(5)		-O1	(x1)	1.864(4)
	-O6a	(x1)	2.521(5)		-O11b	(x1)	1.870(5)
	-O11c	(x1)	2.570(3)		-O13b	(x1)	2.107(9)
	-O13a	(x1)	2.587(8)	BVS for Ga3a1			3.30
	-O3b	(x1)	2.593(5)				
	-O6b	(x1)	2.613(4)	Ga3a2	-O8b	(x1)	1.802(4)
	-O11d	(x1)	2.748(5)		-O1	(x1)	1.820(5)
BVS for La/Ca3b			2.44/1.40		-O11d	(x1)	1.868(3)
					-O11c	(x1)	1.888(4)
La/Ca4a	-O4b	(x1)	2.319(6)		-O13a	(x1)	2.127(9)
	-O12a	(x1)	2.403(5)	BVS for Ga3a2			3.29
	-O8a	(x1)	2.516(3)				
	-O14a	(x1)	2.551(5)	Ga3b1	-O7a	(x1)	1.805(4)
	-O5a	(x1)	2.579(3)		-O12a	(x1)	1.853(5)
	-O5b	(x1)	2.609(4)		-O12b	(x1)	1.855(5)
	-O12b	(x1)	2.657(6)		-O2	(x1)	1.862(3)
	-O4a	(x1)	2.675(9)		-O14a	(x1)	2.177(6)
	-O13b	(x1)	2.777(8)	BVS for Ga3b1			3.28
	-O10d	(x1)	3.032(4)				
	-O10c	(x1)	3.059(4)	Ga3b2	-O7b	(x1)	1.809(4)
BVS for La/Ca4a			2.85/1.64		-O2	(x1)	1.823(4)
					-O12d	(x1)	1.866(3)
La/Ca4b	-O14b	(x1)	2.276(11)		-O12c	(x1)	1.878(5)
	-O4a	(x1)	2.352(7)		-O14b	(x1)	2.143(13)
	-O8b	(x1)	2.476(4)	BVS for Ga3b2			3.28
	-O12c	(x1)	2.519(2)				
	-O12d	(x1)	2.551(5)	O3a	-O3b	(x1)	0.670(7)
	-O13a	(x1)	2.554(8)	O4a	-O4b	(x1)	0.495(9)
	-O5a	(x1)	2.581(4)				
	-O5b	(x1)	2.608(4)	O13a	-O4a	(x1)	1.894(12)
	-O4b	(x1)	2.720(4)	O13b	-O4b	(x1)	1.827(6)
	-O10b	(x1)	2.746(4)	O14a	-O3b	(x1)	1.922(7)
	-O10a	(x1)	2.951(5)	O14b	-O3a	(x1)	1.895(13)
BVS for La/Ca4b			2.78/1.60				

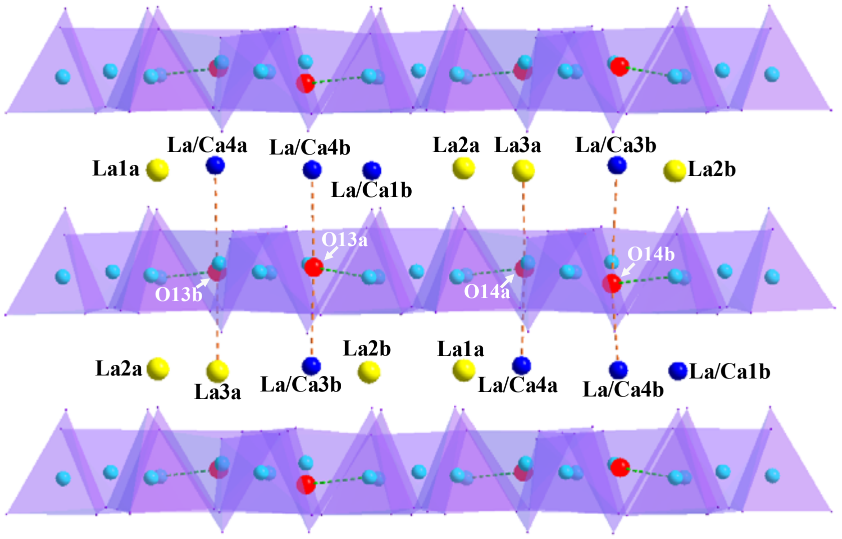
La1a
La/Ca4a
La/Ca4b
La/Ca1b
La2a
La3a
La/Ca3b
La2b
O13a
O13b
O14a
O14b
La2a
La3a
La/Ca3b
La2b
La1a
La/Ca4a
La/Ca4b
La/Ca1b

(a)

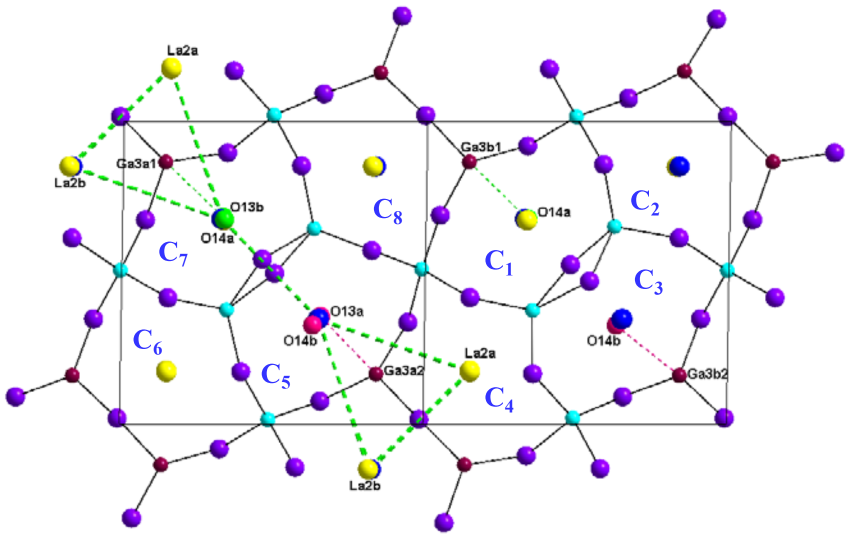
La2a
La2b
Ga3a1
O13b
O14a
Ga3b1
O14a
C8
C7
C1
C2
C3
C6
C5
C4
O13a
O14b
Ga3a2
La2a
O14b
Ga3b2
La2b

(b)

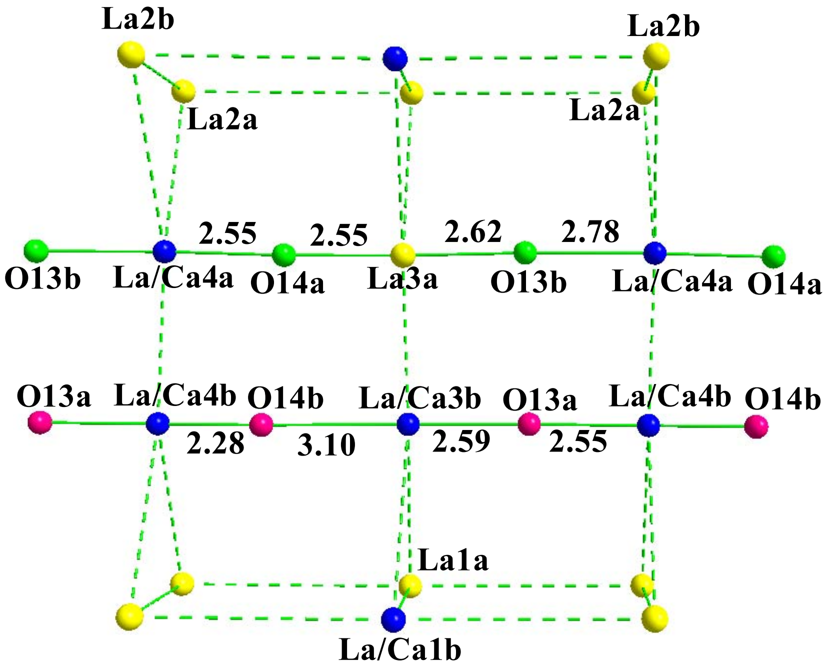

(c)

Figure S5.7 (a) Parallel polyhedral view and (b) projection view along the [110] direction of $La_{1.64}Ca_{0.36}Ga_3O_{7.32}$ (*P1*, average model) to show the A site cation location and ordering. The A site cations fill the Ga_3O_7 interlayer space and sandwich the interstitial sites in the 5-rings. (c) Parallel view of the highlighted part in (b) to show ordered cations and their distances to interstitial oxide, where the Ga and framework oxygens were omitted for clarity. Paired interstitials O13a/O14b and O13b/O14a are shown as red and green spheres in (b) and (c), respectively. La-sites, yellow; La/Ca-sites, blue; La/Ca1b = 0.814(1)/0.186(1), La/Ca3b = 0.312(1)/0.688(1), La/Ca4a = 0.623(1)/0.377(1), La/Ca4b = 0.811(1)/0.189(1).

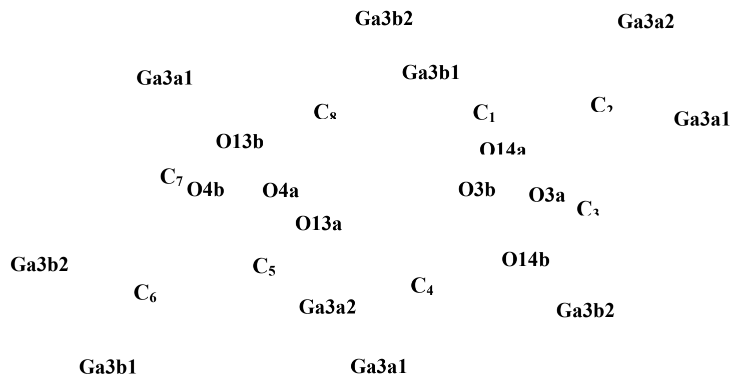

(a)

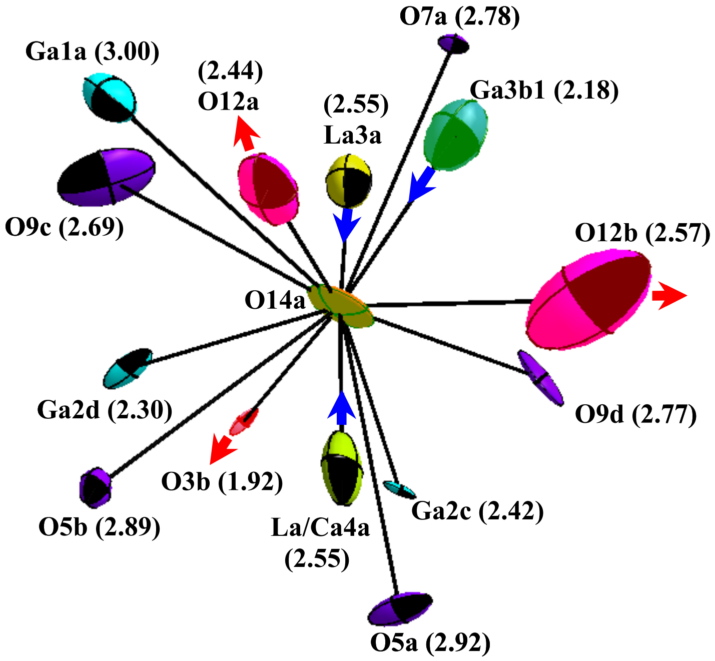

(b)

Figure S5.8 Projection view of $La_{1.64}Ca_{0.36}Ga_3O_{7.32}$ in *P1* along [110] direction to show (a) the location of interstitial oxides and atomic displacement parameters (ADPs, 80% probability shown) within the rings and (b) the environment of O14a within 3Å in the average structure. The numbers denote the distances from each atom to O14a. Local atomic displacements of La3a, La/Ca4a, O3b, O12a, and O12b towards $La3a_L$, $La/Ca4a_L$, O3a, $O12a_L$, and $O12b_L$ due to the O14a incorporation are marked by arrows.

6. Simulated annealing (SA) approach

To validate the structure model (*P1*) from the least-squares Rietveld refinement, simulated annealing (SA) analysis was performed. Peak shape and background parameters were obtained by Le Bail refinement of the NPD data from the 90º and backscattering banks of detectors of the HRPD instrument using TOPAS[3], with a single main melilite phase. The standard simulated annealing macro implemented within TOPAS was run using a fixed framework model from the GSAS Rietveld refinement, with eight possible interstitial oxygen sites specified. The occupancies of these sites were allowed to vary during the minimization, along with the La and Ca occupancies of the split A sites. Penalty functions were specified to bias the total interstitial oxygen and La/Ca occupancies towards previously obtained values constrained by the chemical composition. The SA result (R_{wp} = 6.78 %, R_p = 7.72 %, χ^2 = 4.32, Figure S5.2) is in good agreement with refined structure (Figure S5.1 and Table S5.2) and confirmed the reliability of the proposed structural model regarding the interstitial occupancies and A site cation ordering.

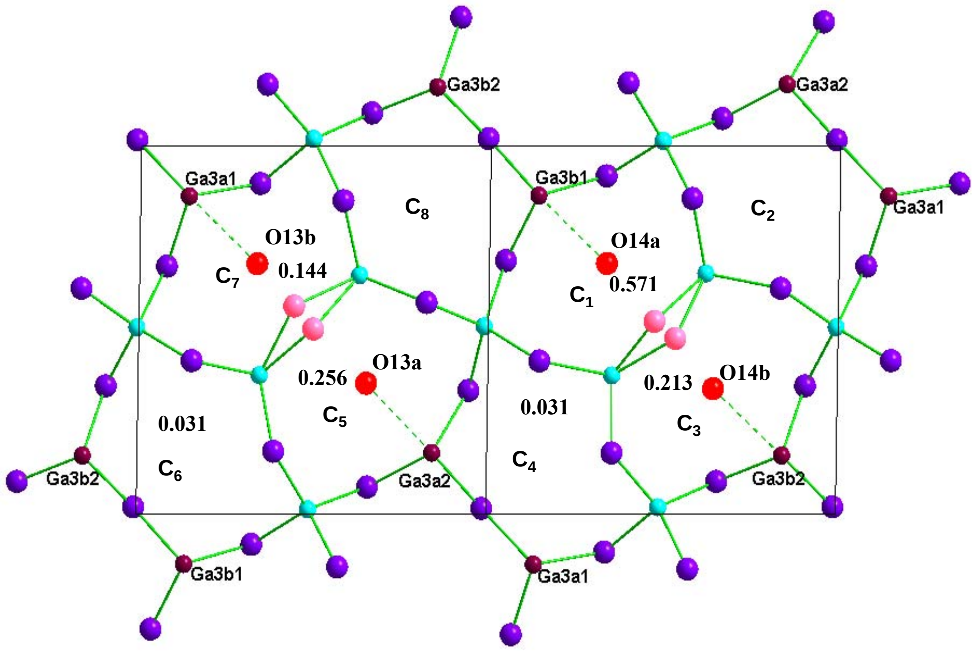

Figure S6.1 Interstitial oxide location and population from the simulated annealing analysis with TOPAS

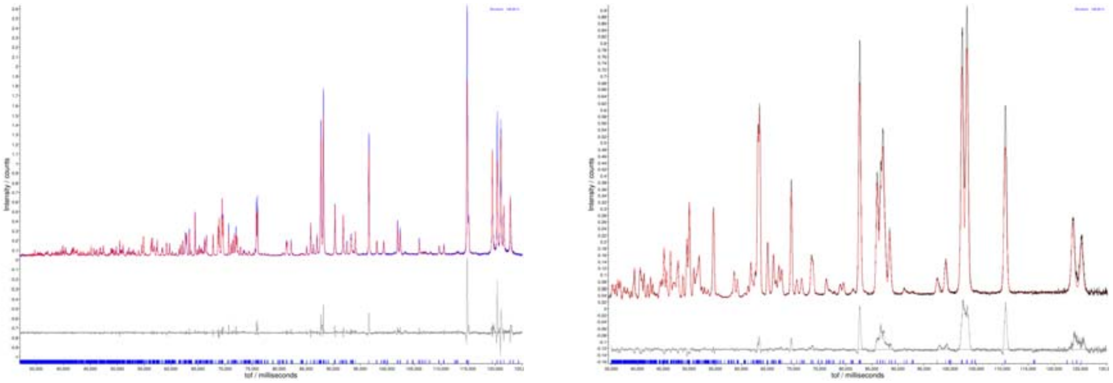

Figure S6.2 SA plots for neutron data from backscattering (left) and 90° bank (right), respectively.

Table S6.1 A-site La/Ca cation ordering extracted from the crystal structure from different approaches (1% error of occupancy), a) GSAS Rietveld refinement (La/Ca = 6.56/1.44); b) TOPAS simulated annealing (La/Ca = 6.61/1.39).

	La/Ca1a	La/Ca1b	La/Ca2a	La/Ca2b	La/Ca3a	La/Ca3b	La/Ca4a	La/Ca4b
a	1.00/000	0.81/0.19	1.00/0.00	1.00/0.00	1.00/0.00	0.31/0.69	0.62/0.38	0.81/0.19
b	0.99/0.01	0.91/0.09	1.00/0.00	0.94/0.06	1.00/0.00	0.35/0.65	0.60/0.40	0.82/0.18

7. Structure refinement for $La_{1.64}Ca_{0.36}Ga_3O_{7.32}$ in split model (triclinic *P1*)

In this split-site model, the relaxations around interstitials were modelled by introducing atomic displacements as shown in Table S7.1-3. Ga3a1, O11a, O11b, and La3a were displaced to $Ga3a1_L$, $O11a_L$, $O11b_L$, and $La3a_S$ around O13b, including O4a in the local defect structure. Similarly, $Ga3a2_L$, $O11c_L$, $O11d_L$, $La/Ca3b_L$, $La/Ca4b_L$, and O4b are involved in the local defect structure around O13a, $Ga3b1_L$, $O12a_L$, $O12b_L$, $La/Ca3a_L$, $La/Ca4a_L$, and O3a around O14a, $Ga3b2_L$, $O12c_L$, $O12d_L$, $La/Ca4b_S$, and O3b around O14b, respectively. This models the cases where an interstitial defect is present and where it is absent.

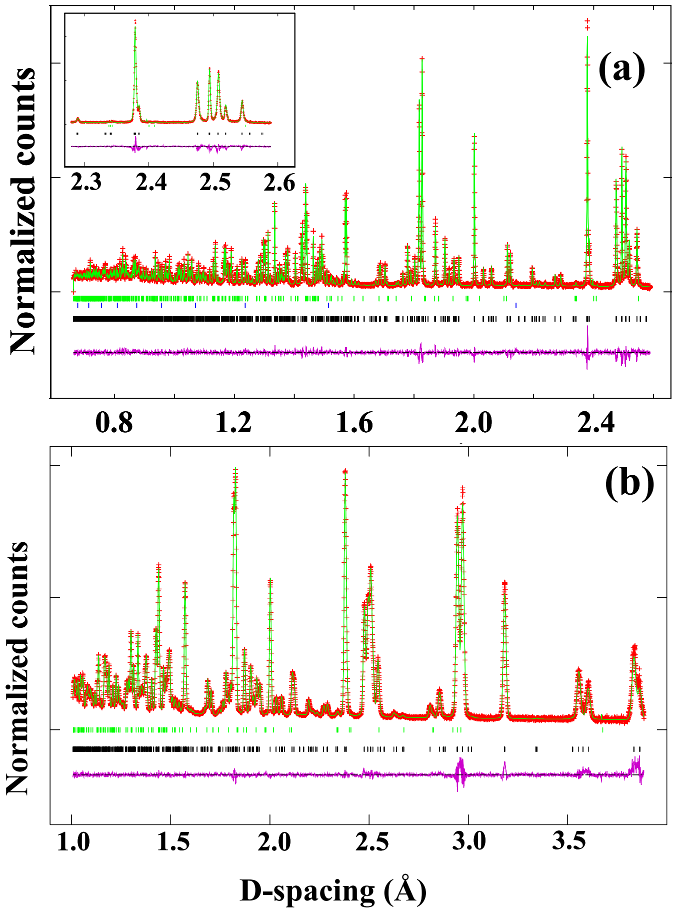

Figure S7.1 Rietveld refinement of RT HRPD ND data for the split-site model for $La_{1.64}Ca_{0.36}Ga_3O_{7.32}$ in *P1* from (a) backscattering bank and (b) 90 degree bank. Three rows of vertical tick mark the reflections from $La_{1.64}Ca_{0.36}Ga_3O_{7.32}$, the vanadium container, and Ga_2O_3 from bottom to top, respectively.

Table S7.1 Refined structural parameters for $La_{1.64}Ca_{0.36}Ga_3O_{7.32}$ at room temperature for the split-site model in the triclinic cell (*P1*)

Atom	*x*	*y*	*z*	U_{iso} (Å^2)	Occupancy
La1a	0.2389(3)	0.0887(2)	0.8285(3)	-	1
La/Ca1b	0.2381(2)	0.4102(3)	0.1501(2)	-	0.825(1)/0.175(1)[a]
La2a	0.7381(4)	0.5852(3)	0.8259(4)	-	1
La2b	0.7343(2)	0.9124(3)	0.1514(2)	-	1
La3a	0.5747(11)	0.2569(8)	0.3257(10)	0.0411(6)	0.241(1)[b]
La3a$_L$	0.5747(5)	0.2394(5)	0.3306(5)	0.0411(6)	0.582(1)[b,3]
La3a$_S$	0.5896(13)	0.2644(14)	0.3227(14)	0.0411(6)	0.177(1)[b,2]
La/Ca3b	0.9059(4)	0.2661(4)	0.6588(4)	0.0027(5)	0.227(1)/0.501(1)[b]
La3/Cab$_L$	0.9158(10)	0.2669(10)	0.6426(10)	0.0027(5)	0.085(1)/0.187(1)[b,1]

La/Ca4a	0.0758(7)	0.7556(7)	0.3202(7)	0.0118(5)	0.260(1)/0.157(1)[b]
La/Ca4a$_L$	0.0886(4)	0.7659(5)	0.3173(5)	0.0118(5)	0.363(1)/0.220(1)[b,3]
La/Ca4b	0.4020(1)	0.7539(1)	0.6549(1)	0.0127(5)	0.375(1)/0.100(1)[b]
La/Ca4b$_L$	0.3808(9)	0.7289(10)	0.649(1)	0.0127(5)	0.220(1)/0.051(1)[b,1]
La/Ca4b$_S$	0.4121(1)	0.7643(1)	0.6548(1)	0.0127(5)	0.199(1)/0.046(1)[b,4]
Ga1a	0.2302(4)	0.7429(3)	0.9842(3)	-	1
Ga1b	0.2353(4)	0.2495(5)	0.4926(4)	-	1
Ga1c	0.7337(4)	0.2434(3)	0.9842(4)	-	1
Ga1d	0.7373(4)	0.7481(4)	0.4931(4)	-	1
Ga2a	0.0023(3)	0.6538(3)	0.6262(3)	-	1
Ga2b	0.0053(3)	0.3810(3)	0.3494(3)	-	1
Ga2c	0.5013(2)	0.1549(2)	0.6210(2)	-	1
Ga2d	0.5006(2)	0.8853(3)	0.3501(3)	-	1
Ga3a1	0.6144(2)	0.4834(3)	0.1359(3)	0.0028(4)	0.823(1)[b]
Ga3a1$_L$	0.6366(4)	0.4876(3)	0.1548(3)	0.0028(4)	0.177(1)[b,2]
Ga3a2	0.3251(3)	0.4794(3)	0.8403(3)	0.0135(5)	0.729(1)[b]
Ga3a2$_L$	0.3073(4)	0.4826(4)	0.8215(5)	0.0135(5)	0.271(1)[b,1]
Ga3b1	0.1140(4)	0.9782(3)	0.1257(4)	0.0105(4)	0.418(1)[b]
Ga3b1$_L$	0.1314(3)	0.9838(3)	0.1438(2)	0.0105(4)	0.582(1)[b,3]
Ga3b2	0.8262(3)	0.9852(3)	0.8482(3)	0.0238(5)	0.755(1)[b]
Ga3b2$_L$	0.8116(4)	0.9844(4)	0.8281(4)	0.0238(5)	0.245(1)[b,4]
O1	0.3945(3)	0.4098(4)	0.9902(3)	-	1
O2	0.8923(3)	0.8988(3)	0.9824(3)	-	1
O3a	0.6158(4)	0.0934(4)	0.5246(4)	-	0.645(6)[c]
O3b	0.5634(6)	0.1024(6)	0.4654(6)	-	0.355(6)[c]
O4a	0.0860(12)	0.5971(12)	0.4910(12)	-	0.157(7)[c]
O4b	0.0396(4)	0.5909(4)	0.4463(4)	-	0.843(7)[c]
O5a	0.3384(3)	0.9802(3)	0.6286(3)	-	1
O5b	0.3343(3)	0.7086(3)	0.3516(3)	-	1
O6a	0.8374(3)	0.4824(3)	0.6339(3)	-	1
O6b	0.8429(3)	0.2076(3)	0.3571(3)	-	1
O7a	0.2889(3)	0.1587(2)	0.1307(2)	-	1
O7b	0.0052(3)	0.1542(3)	0.8507(3)	-	1
O8a	0.7797(3)	0.6522(3)	0.1232(3)	-	1
O8b	0.5078(3)	0.6625(3)	0.8650(3)	-	1
O9a	0.6688(4)	0.3144(5)	0.8340(4)	-	1
O9b	0.7051(5)	0.8742(5)	0.3911(5)	-	1
O9c	0.4649(4)	0.8869(4)	0.1437(4)	-	1
O9d	0.4659(3)	0.3170(4)	0.5778(4)	-	1
O10a	0.2092(4)	0.8201(3)	0.8299(3)	-	1
O10b	0.2237(4)	0.4015(4)	0.4210(4)	-	1
O10c	0.9655(4)	0.3805(5)	0.1486(4)	-	1
O10d	0.9667(3)	0.8159(3)	0.5881(4)	-	1

O11a	0.6190(4)	0.2813(3)	0.0961(5)	0.0453(13)	0.823(1)[b]
O11a$_L$	0.5777(6)	0.2560(5)	0.0496(9)	0.0453(13)	0.177(1)[b,2]
O11b	0.5872(3)	0.5166(5)	0.3255(3)	0.0155(9)	0.823(1)[b]
O11b$_L$	0.5580(5)	0.5326(13)	0.3135(4)	0.0155(9)	0.177(1)[b,2]
O11c	0.1701(4)	0.5185(4)	0.9060(4)	0.0087(7)	0.729(1)[b]
O11c$_L$	0.2004(5)	0.5327(6)	0.9464(6)	0.0087(7)	0.271(1)[b,1]
O11d	0.1791(5)	0.2578(4)	0.6579(4)	0.0136(8)	0.729(1)[b]
O11d$_L$	0.1995(12)	0.2440(8)	0.6682(8)	0.0136(8)	0.271(1)[b]
O12a	0.1210(6)	0.7819(4)	0.1003(7)	0.0243(12)	0.418(1)[b,1]
O12a$_L$	0.0653(4)	0.7473(4)	0.0355(5)	0.0243(12)	0.582(1)[b,3]
O12b	0.1112(6)	0.0199(8)	0.3305(4)	0.0148(10)	0.418(1)[b]
O12b$_L$	0.0678(4)	0.0352(5)	0.3128(4)	0.0148(10)	0.582(1)[b,3]
O12c	0.6620(3)	0.0147(3)	0.8920(4)	0.0037(7)	0.755(1)[b]
O12c$_L$	0.6854(5)	0.0286(6)	0.9398(5)	0.0037(7)	0.245(1)[b,4]
O12d	0.6683(4)	0.7692(4)	0.6517(4)	0.0095(9)	0.755(1)[b]
O12d$_L$	0.7090(7)	0.7465(4)	0.6809(5)	0.0095(9)	0.245(1)[b,4]
O13a	0.1511(7)	0.4990(7)	0.6405(7)	-	0.271(1)[d,1]
O13b	0.8136(4)	0.5007(6)	0.3454(6)	-	0.177(1)[d,2]
O14a	0.3330(4)	1.0134(4)	0.3316(4)	-	0.582(1)[d,3]
O14b	0.6262(6)	0.9658(5)	0.6346(4)	-	0.245(1)[d,4]

R_{wp}/R_p%(ND168) = 6.32/5.99, R_{wp}/R_p%(ND90) = 2.83/3.41, R_{wp}/R_p%(Total)= 3.30/4.72, χ^2 = 4.81.

[a] Occupancies of mixed La/Ca sited were constrained to be unity, the ADPs were refined to have the same values.

[b] ADPs for related bulk- and defect- structure atoms were constrained to be the same, and the total occupancy was constrained to be unity, e.g. Occ.(La3a) + Occ.(La3a$_L$) + Occ.(La3a$_S$) = 1.

[c] Occupancies of O3a/O3b, and O4a/O4b pairs were constrained to be unity, and the ADPs were refined to have the same values, respectively.

[d] The interstitial oxide content was fixed according to the charge-balanced nominal composition of 1.28 in the unit cell.

[1,2,3,4] Occupancies were constrained to have the same values as the defect interstitials with which they are associated.

**Atom**	**U_{11} (Å^2)**	**U_{22} (Å^2)**	**U_{33} (Å^2)**	**U_{12} (Å^2)**	**U_{13}(Å^2)**	**U_{23} (Å^2)**
La1a	0.0094(6)	0.0083(6)	0.0101(7)	0.0085(5)	0.0033(5)	0.0025(6)
La/Ca1b[a]	0.0067(7)	0.0073(7)	0.0058(7)	0.0047(6)	0.0018(6)	0.0042(6)
La2a	0.0406(9)	0.0485(12)	0.0465(12)	0.0287(8)	0.0213(7)	0.0109(10)
La2b	0.0160(7)	0.0254(8)	0.0195(9)	0.0126(6)	0.0110(6)	0.0089(7)

Ga1a	0.0119(8)	0.0112(8)	0.0153(8)	0.0039(6)	0.0056(7)	0.0083(7)
Ga1b	0.0163(7)	0.0139(8)	0.0127(8)	0.0068(5)	0.0108(6)	0.0050(8)
Ga1c	0.0116(7)	0.0141(8)	0.0189(9)	0.0102(5)	0.0042(7)	0.0094(8)
Ga1d	0.0069(7)	0.0044(7)	0.0060(5)	0.0042(5)	0.0011(6)	0.0033(7)
Ga2a	0.0156(8)	0.0256(10)	0.0256(10)	0.0112(7)	0.0128(6)	0.0052(10)
Ga2b	0.0298(10)	0.0392(12)	0.0350(11)	0.0193(9)	0.0201(7)	0.0099(11)
Ga2c	0.0013(6)	0.0015(6)	0.0021(7)	0.0010(5)	0.0005(5)	0.0009(7)
Ga2d	0.0116(8)	0.0232(10)	0.0174(9)	0.0071(8)	0.0129(6)	0.0064(9)
O1	0.0289(11)	0.0313(12)	0.0290(12)	0.0146(8)	0.0161(9)	0.0121(10)
O2	0.0096(9)	0.0134(9)	0.0095(9)	0.0030(7)	0.0079(7)	0.0069(8)
O3a/3b[a]	0.0061(11)	0.0037(13)	0.0031(12)	-0.001(1)	0.0031(8)	-0.001(1)
O4a/4b[a]	0.0423(16)	0.0468(19)	0.0397(18)	0.0109(16)	0.0217(12)	0.0163(13)
O5a	0.0132(12)	0.0144(13)	0.0150(13)	0.0038(10)	0.0051(9)	-0.001(1)
O5b	0.0224(13)	0.0228(14)	0.0263(14)	0.0102(11)	0.0135(9)	0.0135(12)
O6a	0.0042(10)	0.0062(11)	0.0078(11)	0.0021(8)	0.0034(8)	-0.001(1)
O6b	0.0161(12)	0.0169(12)	0.0196(12)	0.0065(10)	0.0078(9)	0.0179(10)
O7a	0.0022(9)	0.0018(9)	0.0021(10)	0.0015(7)	0.0009(8)	0.0016(8)
O7b	0.0239(13)	0.0170(11)	0.0243(12)	0.009(1)	0.0096(11)	0.0195(8)
O8a	0.0151(12)	0.0168(14)	0.0243(14)	0.0041(10)	0.0091(11)	0.0065(10)
O8b	0.0186(11)	0.0212(11)	0.0338(14)	0.0175(9)	0.0108(11)	0.0185(10)
O9a	0.0463(15)	0.0380(17)	0.0393(19)	0.0303(11)	0.0209(14)	-0.007(2)
O9b	0.0693(18)	0.071(2)	0.089(3)	0.0565(13)	0.0456(19)	0.016(2)
O9c	0.0079(11)	0.0316(16)	0.0228(15)	0.0096(11)	0.0085(11)	0.0081(14)
O9d	0.0031(11)	0.0130(12)	0.0152(12)	-0.001(1)	-0.001(1)	0.0123(11)
O10a	0.0306(15)	0.0181(13)	0.0222(15)	0.0135(11)	0.0009(14)	0.0161(11)
O10b	0.0154(13)	0.0138(12)	0.0113(12)	0.0053(10)	0.0013(11)	0.0113(10)
O10c	0.0215(13)	0.051(2)	0.0231(16)	0.0127(14)	0.0161(11)	0.0046(17)
O10d	0.0115(11)	0.0142(12)	0.0246(16)	0.0121(8)	0.0014(12)	0.0071(13)
O13a/13b[a]	0.009(3)	0.006(4)	0.023(4)	-0.002(2)	0.008(3)	-0.009(3)
O14a	0.0088(18)	0.0042(18)	0.0030(18)	0.0011(14)	0.0030(15)	0.0027(12)
O14b	0.34(4)	0.21(3)	0.37(4)	0.04(2)	0.11(2)	-0.21(2)

[a] ADPs for La/Ca on all sites and for O3a/O3b, O4a/O4b, O13a and O13b were constrained to be the same value.

Table S7.2 Selected inter-atomic distances and BVS calculations for $La_{1.64}Ca_{0.36}Ga_3O_{7.32}$ at room temperature from the split-site model in *P1*.

**Bulk Structure**				**Defect Structure**			
**Bond**			**Length (Å)**	**Bond**			**Length (Å)**
La1a	-O10a	(x1)	2.476(4)	La1a	-O11d_L	(x1)	2.471(9)
	-O1	(x1)	2.495(4)		-O11a_L	(x1)	2.679(5)

	-O10d	(x1)	2.503(3)				
	-O5a	(x1)	2.534(4)				
	-O7b	(x1)	2.605(4)				
	-O7a	(x1)	2.608(3)				
	-O11d	(x1)	2.701(5)				
	-O11a	(x1)	3.051(4)				
BVS for La1a			2.59				
La1b	-O1	(x1)	2.452(5)	La1b	-O11b$_L$	(x1)	2.507(5)
	-O10c	(x1)	2.505(5)		-O11c$_L$	(x1)	2.554(7)
	-O5b	(x1)	2.518(4)				
	-O7b	(x1)	2.605(3)				
	-O7a	(x1)	2.607(4)				
	-O10b	(x1)	2.662(5)				
	-O11b	(x1)	2.797(4)				
	-O11c	(x1)	2.818(5)				
BVS for La1b			2.51				
La2a	-O2	(x1)	2.434(4)	La2a	-O12d$_L$	(x1)	2.393(6)
	-O9a	(x1)	2.438(6)		-O12a$_L$	(x1)	2.579(4)
	-O6a	(x1)	2.459(5)				
	-O9d	(x1)	2.511(3)				
	-O8a	(x1)	2.589(5)				
	-O8b	(x1)	2.684(5)				
	-O12d	(x1)	2.875(6)				
	-O12a	(x1)	3.084(5)				
BVS for La2a			2.65				
La2b	-O9c	(x1)	2.467(5)	La2b	-O12c$_L$	(x1)	2.585(7)
	-O6b	(x1)	2.472(4)		-O12b$_L$	(x1)	2.615(4)
	-O9b	(x1)	2.505(6)				
	-O8b	(x1)	2.511(3)				
	-O2	(x1)	2.546(4)				
	-O8a	(x1)	2.649(5)				
	-O12c	(x1)	2.911(5)				
	-O12b	(x1)	3.029(6)				
BVS for La2b			2.58				
La3a	-O3b	(x1)	2.256(12)	La3a	-O13b	(x1)	2.436(10)
	-O7a	(x1)	2.367(9)		-O14a	(x1)	2.578(10)
	-O11a	(x1)	2.421(12)		-O11a$_L$	(x1)	2.649(14)
	-O11b	(x1)	2.441(10)		-O11b$_L$	(x1)	2.740(16)
	-O6a	(x1)	2.655(7)				

	-O6b	(x1)	2.733(12)	La3a$_L$	-O7a	(x1)	2.437(5)
	-O3a	(x1)	2.823(11)		-O14a	(x1)	2.463(6)
	-O9d	(x1)	2.912(12)		-O6b	(x1)	2.647(6)
	-O9c	(x1)	3.002(8)		-O3a	(x1)	2.653(7)
BVS for La3a			2.71		-O6a	(x1)	2.654(4)
					-O11a$_L$	(x1)	2.747(10)
					-O9c	(x1)	2.864(6)
					-O9d	(x1)	2.893(7)
					-O11b$_L$	(x1)	2.922(14)
				BVS for La3a$_L$			2.31/1.33
				La3a$_S$	-O13b	(x1)	2.306(13)
					-O3b	(x1)	2.336(16)
					-O7a	(x1)	2.447(11)
					-O11a$_L$	(x1)	2.556(17)
					-O6a	(x1)	2.628(9)
					-O6b	(x1)	2.634(15)
					-O14a	(x1)	2.712(13)
					-O11b$_L$	(x1)	2.725(20)
					-O3a	(x1)	2.886(16)
					-O9c	(x1)	3.021(13)
					-O9d	(x1)	3.030(15)
				BVS for La3a$_S$			2.85/1.64
La/Ca3b	-O3a	(x1)	2.232(4)	La/Ca3b	-O13a	(x1)	2.611(8)
	-O11c	(x1)	2.434(3)		-O11c$_L$	(x1)	2.721(4)
	-O6a	(x1)	2.458(5)		-O11d$_L$	(x1)	2.877(13)
	-O7b	(x1)	2.507(5)		-O14b	(x1)	2.929(6)
	-O6b	(x1)	2.593(5)				
	-O11d	(x1)	2.650(7)				
	-O3b	(x1)	2.659(6)	La/Ca3b$_L$	-O3a	(x1)	2.317(8)
BVS for La/Ca3b			2.60/1.50		-O6b	(x1)	2.423(10)
					-O13a	(x1)	2.464(11)
					-O6a	(x1)	2.473(12)
					-O7b	(x1)	2.603(11)
					-O3b	(x1)	2.718(9)
					-O11dL	(x1)	2.752(16)
					-O11cL	(x1)	2.758(7)
				BVS for La/Ca3b$_L$			2.65/1.52
La/Ca4a	-O4b	(x1)	2.233(9)	La/Ca4a	-O14a	(x1)	2.645(7)
	-O12a	(x1)	2.338(10)		-O12a$_L$	(x1)	2.672(9)
	-O12b	(x1)	2.383(11)		-O12b$_L$	(x1)	2.728(9)

	-O8a	(x1)	2.432(6)		-O13b	(x1)	2.854(8)
	-O4a	(x1)	2.536(15)				
	-O5b	(x1)	2.630(8)	La/Ca4a$_L$	-O4b	(x1)	2.341(7)
	-O5a	(x1)	2.655(5)		-O8a	(x1)	2.493(4)
	-O10c	(x1)	3.032(8)		-O14a	(x1)	2.510(5)
	-O10d	(x1)	3.049(9)		-O5b	(x1)	2.567(6)
BVS for La/Ca4a			3.23/1.82		-O12a$_L$	(x1)	2.583(7)
					-O5a	(x1)	2.630(4)
					-O4a	(x1)	2.630(14)
					-O12b$_L$	(x1)	2.675(7)
					-O10c	(x1)	3.084(6)
				BVS for La/Ca4a$_L$			2.71/1.55
La/Ca4b	-O12c	(x1)	2.414(2)	La/Ca4b	-O14b	(x1)	2.403(5)
	-O4a	(x1)	2.433(9)		-O13a	(x1)	2.582(6)
	-O12d	(x1)	2.505(4)		-O12c$_L$	(x1)	2.695(3)
	-O8b	(x1)	2.507(4)		-O12d$_L$	(x1)	2.903(7)
	-O5a	(x1)	2.530(4)				
	-O5b	(x1)	2.629(3)				
	-O10b	(x1)	2.823(3)	La/Ca4b$_L$	-O13a	(x1)	2.369(11)
	-O4b	(x1)	2.831(3)		-O8b	(x1)	2.441(11)
	-O10a	(x1)	2.976(4)		-O5a	(x1)	2.644(11)
BVS for La/Ca4b			2.51/1.44		-O10b	(x1)	2.663(8)
					-O5b	(x1)	2.675(11)
					-O4b	(x1)	2.702(8)
					-O12c$_L$	(x1)	2.852(6)
					-O10a	(x1)	2.916(11)
					-O12dL	(x1)	2.990(12)
				BVS for La/Ca4b$_L$			2.48/1.42
				La/Ca4b$_S$	-O14b	(x1)	2.295(5)
					-O5a	(x1)	2.487(4)
					-O4a	(x1)	2.505(9)
					-O8b	(x1)	2.555(4)
					-O5b	(x1)	2.589(3)
					-O12c$_L$	(x1)	2.640(3)
					-O12d$_L$	(x1)	2.853(7)
					-O10b	(x1)	2.885(3)
					-O4b	(x1)	2.895(3)
					-O10a	(x1)	3.034(4)
				BVS for La/Ca4b$_S$			2.69/1.55
Ga1a	-O12a	(x1)	1.806(8)	Ga1a	-O12a$_L$	(x1)	1.805(6)

	-O11c	(x1)	1.823(5)		-O11c$_L$	(x1)	1.822(7)
	-O10a	(x1)	1.824(5)		-O10a	(x1)	1.824(5)
	-O9c	(x1)	1.858(4)		-O9c	(x1)	1.858(4)
BVS for Ga1a			3.08	BVS for Ga1a			3.08
Ga1b	-O10b	(x1)	1.804(7)	Ga1b	-O10b	(x1)	1.804(7)
	-O11d	(x1)	1.814(6)		-O11d$_L$	(x1)	1.828(10)
	-O9d	(x1)	1.841(5)		-O12b$_L$	(x1)	1.832(4)
	-O12b	(x1)	1.857(6)		-O9d	(x1)	1.841(5)
BVS for Ga1b			3.07	BVS for Ga1b			3.09
Ga1c	-O9a	(x1)	1.805(6)	Ga1c	-O12c$_L$	(x1)	1.799(7)
	-O11a	(x1)	1.819(7)		-O9a	(x1)	1.805(6)
	-O12c	(x1)	1.831(4)		-O11a$_L$	(x1)	1.822(9)
	-O10c	(x1)	1.852(4)		-O10c	(x1)	1.852(4)
BVS for Ga1c			3.08	BVS for Ga1c			3.14
Ga1d	-O9b	(x1)	1.815(7)	Ga1d	-O9b	(x1)	1.815(7)
	-O12d	(x1)	1.826(6)		-O10d	(x1)	1.829(5)
	-O10d	(x1)	1.829(5)		-O11b$_L$	(x1)	1.856(7)
	-O11b	(x1)	1.876(4)		-O12d$_L$	(x1)	1.903(7)
BVS for Ga1d			3.01	BVS for Ga1d			2.90
Ga2a	-O4a	(x1)	1.788(13)				
	-O6a	(x1)	1.807(4)				
	-O10d	(x1)	1.824(5)				
	-O4b	(x1)	1.846(5)				
	-O10a	(x1)	1.882(3)				
	-O13b	(x1)	2.284(5)				
BVS for Ga2a			3.00				
Ga2b	-O4a	(x1)	1.779(10)				
	-O4b	(x1)	1.792(5)				
	-O6b	(x1)	1.806(4)				
	-O10c	(x1)	1.836(5)				
	-O10b	(x1)	1.892(5)				
BVS for Ga2b			3.06				
Ga2c	-O3b	(x1)	1.784(7)				
	-O3a	(x1)	1.798(5)				
	-O9a	(x1)	1.808(3)				
	-O5a	(x1)	1.815(4)				
	-O9d	(x1)	1.843(5)				

BVS for Ga2c			3.13				
Ga2d	-O3a	(x1)	1.775(3)				
	-O3b	(x1)	1.779(6)				
	-O5b	(x1)	1.810(4)				
	-O9c	(x1)	1.902(5)				
	-O9b	(x1)	1.926(6)				
BVS for Ga2d			2.90				
Ga3a1	-O1	(x1)	1.814(3)	Ga3a1	-O1	(x1)	1.814(3)
	-O8a	(x1)	1.823(4)		-O8a	(x1)	1.823(4)
	-O11b	(x1)	1.868(4)		-O11a$_L$	(x1)	1.927(6)
	-O11a	(x1)	1.882(5)		-O11b$_L$	(x1)	1.927(6)
BVS for Ga3a1			2.93		-O13b	(x1)	2.203(6)
				BVS for Ga3a1			3.03
				Ga3a1$_L$	-O8a	(x1)	1.806(4)
					-O11a$_L$	(x1)	1.899(6)
					-O11b$_L$	(x1)	1.911(6)
					-O13b	(x1)	1.996(6)
					-O1	(x1)	2.019(4)
				BVS for Ga3a1$_L$			3.01
Ga3a2	-O8b	(x1)	1.802(4)	Ga3a2	-O8b	(x1)	1.802(4)
	-O1	(x1)	1.804(5)		-O1	(x1)	1.804(5)
	-O11d	(x1)	1.864(3)		-O11c$_L$	(x1)	1.913(7)
	-O11c	(x1)	1.904(6)		-O11d$_L$	(x1)	1.923(6)
BVS for Ga3a2			2.96		-O13a	(x1)	2.221(8)
				BVS for Ga3a2			3.11
				Ga3a2$_L$	-O8b	(x1)	1.810(4)
					-O11c$_L$	(x1)	1.879(8)
					-O11d$_L$	(x1)	1.917(7)
					-O13a	(x1)	1.997(8)
					-O1	(x1)	2.015(6)
				BVS for Ga3a2$_L$			3.03
Ga3b1	-O2	(x1)	1.805(4)	Ga3b1	-O2	(x1)	1.805(4)
	-O7a	(x1)	1.840(4)		-O7a	(x1)	1.840(4)
	-O12a	(x1)	1.855(6)		-O12a$_L$	(x1)	1.916(5)
	-O12b	(x1)	1.895(6)		-O12b$_L$	(x1)	1.955(6)
BVS for Ga3b1			2.91		-O14a	(x1)	2.206(5)
				BVS for Ga3b1			2.98

				$Ga3b1_L$	-O7a	(x1)	1.822(3)
					$-O12b_L$	(x1)	1.903(5)
					$-O12a_L$	(x1)	1.922(4)
					-O2	(x1)	1.967(3)
					-O14a	(x1)	2.035(4)
				BVS for $Ga3b1_L$			2.97
Ga3b2	-O2	(x1)	1.805(4)	Ga3b2	-O2	(x1)	1.805(4)
	-O7b	(x1)	1.815(4)		-O7b	(x1)	1.815(4)
	-O12c	(x1)	1.849(5)		$-O12c_L$	(x1)	1.931(7)
	-O12d	(x1)	1.894(3)		$-O12d_L$	(x1)	1.941(4)
BVS for Ga3b2			2.98		-O14b	(x1)	2.221(5)
				BVS for Ga3b2			3.02
				$Ga3b2_L$	-O7b	(x1)	1.807(4)
					$-O12d_L$	(x1)	1.907(5)
					$-O12c_L$	(x1)	1.932(7)
					-O2	(x1)	1.985(5)
					-O14b	(x1)	2.029(6)
				BVS for $Ga3b2_L$			2.96
O13a	-O4b	(x1)	2.413(9)	O13b	-O4a	(x1)	2.160(11)
O14a	-O3a	(x1)	2.395(5)	O14b	-O3b	(x1)	2.436(8)

Table S7.3 Bond angles in the GaO_n polyhedra of $La_{1.64}Ca_{0.36}Ga_3O_{7.32}$ from the split-model in *P1*.

**Bulk structure**				**Defect structure**			
**Bond**			**Angle (°)**	**Bond**			**Angle (°)**
	$G1aO_4$ tetrahedron				$G1aO_4$ tetrahedron		
O12a	-Ga1a-	O11c	109.8(3)	$O12a_L$	-Ga1a-	$O11c_L$	106.2(3)
O12a	-Ga1a-	O10a	113.4(2)	$O12a_L$	-Ga1a-	O10a	102.1(2)
O12a	-Ga1a-	O9c	103.5(3)	$O12a_L$	-Ga1a-	O9c	120.8(3)
O11c	-Ga1a-	O10a	115.6(2)	$O11c_L$	-Ga1a-	O10a	125.0(3)
O11c	-Ga1a-	O9c	109.2(2)	$O11c_L$	-Ga1a-	O9c	100.0(2)
O10a	-Ga1a-	O9c	104.4(2)	O10a	-Ga1a-	O9c	104.4(2)
Average			109.3	Average			109.8
	$Ga1bO_4$ tetrahedron				$Ga1bO_4$ tetrahedron		
O10b	-Ga1b-	O11d	113.4(3)	O10b	-Ga1b-	$O11d_L$	121.5(3)

O10b	-Ga1b-	O9d	104.5(3)
O10b	-Ga1b-	O12b	116.6(4)
O11d	-Ga1b-	O9d	108.2(3)
O11d	-Ga1b-	O12b	108.3(2)
O9d	-Ga1b-	O12b	105.1(3)
Average			109.4

O10b	-Ga1b-	$O12b_L$	105.3(3)
O10b	-Ga1b-	O9d	104.5(3)
$O11d_L$	-Ga1b-	$O12b_L$	109.0(3)
$O11d_L$	-Ga1b-	O9d	101.3(4)
$O12b_L$	-Ga1b-	O9d	119.3(3)
Average			109.6

$Ga1cO_4$ tetrahedron

O9a	-Ga1c-	O11a	100.0(2)
O9a	-Ga1c-	O12c	114.4(3)
O9a	-Ga1c-	O10c	116.4(2)
O11a	-Ga1c-	O12c	110.2(2)
O11a	-Ga1c-	O10c	103.6(3)
O12c	-Ga1c-	O10c	110.9(2)
Average			109.3

$Ga1cO_4$ tetrahedron

$O12c_L$	-Ga1c-	O9a	126.8(3)
$O12c_L$	-Ga1c-	$O11a_L$	103.2(3)
$O12c_L$	-Ga1c-	O10c	102.4(3)
O9a	-Ga1c-	$O11a_L$	91.3(3)
O9a	-Ga1c-	O10c	116.4(2)
$O11a_L$	-Ga1c-	O10c	116.2(3)
Average			109.4

$Ga1dO_4$ tetrahedron

O9b	-Ga1d-	O12d	111.6(3)
O9b	-Ga1d-	O10d	110.0(3)
O9b	-Ga1d-	O11b	105.4(3)
O12d	-Ga1d-	O10d	109.9(3)
O12d	-Ga1d-	O11b	106.7(2)
O10d	-Ga1d-	O11b	113.2(2)
Average			109.5

$Ga1dO_4$ tetrahedron

O9b	-Ga1d-	O10d	110.0(3)
O9b	-Ga1d-	$O11b_L$	96.5(3)
O9b	-Ga1d-	$O12d_L$	127.5(3)
O10d	-Ga1d-	$O11b_L$	123.9(3)
O10d	-Ga1d-	$O12d_L$	96.2(3)
$O11b_L$	-Ga1d-	$O12d_L$	105.5(2)
Average			109.9

$Ga2aO_4$ tetrahedron

O4a	-Ga2a-	O6a	118.5(4)
O4a	-Ga2a-	O10d	103.1(4)
O4a	-Ga2a-	O10a	101.4(3)
O6a	-Ga2a-	O10d	120.2(2)
O6a	-Ga2a-	O4b	116.8(2)
O6a	-Ga2a-	O10a	116.0(2)
O10d	-Ga2a-	O4b	92.8(2)
O10d	-Ga2a-	O10a	93.3(2)
O4b	-Ga2a-	O10a	113.4(2)
Average			108.4

$Ga2bO_4$ tetrahedron

O4a	-Ga2b-	O6b	117.8(4)
O4a	-Ga2b-	O10c	104.5(4)
O4a	-Ga2b-	O10b	96.3(4)
O4b	-Ga2b-	O6b	117.0(2)
O4b	-Ga2b-	O10c	93.3(2)

O4b	-Ga2b-	O10b	109.1(2)
O6b	-Ga2b-	O10c	119.6(2)
O6b	-Ga2b-	O10b	114.4(2)
O10c	-Ga2b-	O10b	100.5(2)
Average			108.1

$Ga2cO_4$ tetrahedron

O3b	-Ga2c-	O9a	119.1(2)
O3b	-Ga2c-	O5a	118.7(2)
O3b	-Ga2c-	O9d	94.8(2)
O3a	-Ga2c-	O9a	104.2(2)
O3a	-Ga2c-	O5a	114.1(2)
O3a	-Ga2c-	O9d	112.6(2)
O9a	-Ga2c-	O5a	107.4(2)
O9a	-Ga2c-	O9d	91.9(2)
O5a	-Ga2c-	O9d	122.2(2)
Average			109.5

$Ga2dO_4$ tetrahedron

O3a	-Ga2d-	O5b	117.1(2)
O3a	-Ga2d-	O9c	114.4(2)
O3a	-Ga2d-	O9b	94.0(2)
O3b	-Ga2d-	O5b	121.0(3)
O3b	-Ga2d-	O9c	96.9(2)
O3b	-Ga2d-	O9b	109.7(3)
O5b	-Ga2d-	O9c	119.1(2)
O5b	-Ga2d-	O9b	111.9(2)
O9c	-Ga2d-	O9b	94.5(2)
Average			108.7

$Ga3a1O_4$ tetrahedron

O1	-Ga3a1-	O8a	112.5(2)
O1	-Ga3a1-	O11b	97.4(2)
O1	-Ga3a1-	O11a	105.2(2)
O8a	-Ga3a1-	O11b	123.7(2)
O8a	-Ga3a1-	O11a	116.2(2)
O11b	-Ga3a1-	O11a	98.7(2)
Average			109.0

$Ga3a1O_4$ tetrahedron

O1	-Ga3a1-	O8a	112.5(2)
O1	-Ga3a1-	$O11a_L$	94.0(3)
O1	-Ga3a1-	$O11b_L$	88.5(2)
O8a	-Ga3a1-	$O11a_L$	118.4(2)
O8a	-Ga3a1-	$O11b_L$	122.4(2)
$O11a_L$	-Ga3a1-	$O11b_L$	112.4(3)
Average			108.0

$Ga3a1_LO_4$ trigonal bipyramid

Equatorial planar

O8a	$-Ga3a1_L-$	$O11a_L$	120.8(2)
O8a	$-Ga3a1_L-$	$O11b_L$	124.3(3)

				O11a$_L$	-Ga3a1$_L$-	O11b$_L$	114.4(3)
				Average			119.8
					Apex		
				O13b	-Ga3a1$_L$-	O1	155.8(2)
					Others		
				O8a	-Ga3a1$_L$-	O13b	99.6(2)
				O8a	-Ga3a1$_L$-	O1	104.3(2)
				O11a$_L$	-Ga3a1$_L$-	O1	88.5(3)
				O11b$_L$	-Ga3a1$_L$-	O13b	80.4(2)
				O11b$_L$	-Ga3a1$_L$-	O1	83.2(2)
				Average			89.7
	Ga3a2O$_4$ tetrahedron				Ga3a2O$_4$ tetrahedron		
O8b	-Ga3a2-	O1	111.3(2)	O8b	-Ga3a2-	O1	111.3(2)
O8b	-Ga3a2-	O11d	127.3(2)	O8b	-Ga3a2-	O11c$_L$	115.6(2)
O8b	-Ga3a2-	O11c	117.6(2)	O8b	-Ga3a2-	O11d$_L$	126.4(3)
O1	-Ga3a2-	O11d	95.1(2)	O1	-Ga3a2-	O11c$_L$	87.0(2)
O1	-Ga3a2-	O11c	95.5(2)	O1	-Ga3a2-	O11d$_L$	88.1(3)
O11d	-Ga3a2-	O11c	103.4(2)	O11c$_L$	-Ga3a2-	O11d$_L$	114.8(3)
Average			108.4	Average			107.2
					Ga3a2$_L$O$_4$ trigonal bipyramid		
					Equatorial planar		
				O8b	-Ga3a2$_L$-	O11c$_L$	116.9(3)
				O8b	-Ga3a2$_L$-	O11d$_L$	126.3(3)
				O11c$_L$	-Ga3a2$_L$-	O11d$_L$	116.7(4)
				Average			120.0
					Apex		
				O13a	-Ga3a2$_L$-	O1	162.5(3)
					Others		
				O8b	-Ga3a2$_L$-	O13a	95.4(3)
				O8b	-Ga3a2$_L$-	O1	102.0(2)
				O11c$_L$	-Ga3a2$_L$-	O13a	88.8(3)
				O11c$_L$	-Ga3a2$_L$-	O1	82.1(2)
				O11d$_L$	-Ga3a2$_L$-	O13a	88.3(3)
				O11d$_L$	-Ga3a2$_L$-	O1	82.5(3)
				Average			89.9
	Ga3b1O$_4$ tetrahedron				Ga3b1O$_4$ tetrahedron		
O2	-Ga3b1-	O7a	117.2(2)	O2	-Ga3b1-	O7a	117.2(2)
O2	-Ga3b1-	O12a	105.2(3)	O2	-Ga3b1-	O12a$_L$	89.9(2)
O2	-Ga3b1-	O12b	102.3(2)	O2	-Ga3b1-	O12b$_L$	90.0(2)
O7a	-Ga3b1-	O12a	119.2(2)	O7a	-Ga3b1-	O12a$_L$	123.1(2)

O7a	-Ga3b1-	O12b	115.8(3)	O7a	-Ga3b1-	O12b$_L$	116.4(2)
O12a	-Ga3b1-	O12b	93.4(3)	O12a$_L$	-Ga3b1-	O12b$_L$	112.2(2)
Average			108.9	Average			108.1
				Ga3b1$_L$O$_4$ trigonal bipyramid			
				Equatorial planar			
				O7a	-Ga3b1$_L$-	O12b$_L$	120.0(2)
				O7a	-Ga3b1$_L$-	O12a$_L$	123.8(2)
				O12b$_L$	-Ga3b1$_L$-	O12a$_L$	114.3(2)
				Average			119.4
				Apex			
				O2	-Ga3b1$_L$-	O14a	160.6(2)
				Others			
				O7a	-Ga3b1$_L$-	O2	110.4(2)
				O7a	-Ga3b1$_L$-	O14a	88.9(2)
				O12b$_L$	-Ga3b1$_L$-	O2	86.8(2)
				O12b$_L$	-Ga3b1$_L$-	O14a	82.0(2)
				O12a$_L$	-Ga3b1$_L$-	O2	85.1(2)
				O12a$_L$	-Ga3b1$_L$-	O14a	85.2(2)
				Average			89.7
Ga3b2O$_4$ tetrahedron				Ga3b2O$_4$ tetrahedron			
O2	-Ga3b2-	O7b	114.4(2)	O2	-Ga3b2-	O7b	114.4(2)
O2	-Ga3b2-	O12c	102.8(2)	O2	-Ga3b2-	O12c$_L$	93.3(2)
O2	-Ga3b2-	O12d	95.8(2)	O2	-Ga3b2-	O12d$_L$	80.8(2)
O7b	-Ga3b2-	O12c	122.7(2)	O7b	-Ga3b2-	O12c$_L$	122.8(2)
O7b	-Ga3b2-	O12d	119.3(2)	O7b	-Ga3b2-	O12d$_L$	119.7(2)
O12c	-Ga3b2-	O12d	97.3(2)	O12c$_L$	-Ga3b2-	O12d$_L$	113.2(2)
Average			108.7	Average			107.4
				Ga3b2$_L$O$_4$ trigonal bipyramid			
				Equatorial planar			
				O7b	-Ga3b2$_L$-	O12d$_L$	121.9(3)
				O7b	-Ga3b2$_L$-	O12c$_L$	123.2(3)
				O12d$_L$	-Ga3b2$_L$-	O12c$_L$	114.8(3)
				Average			120.0
				Apex			
				O2	-Ga3b2$_L$-	O14b	152.8(2)
				Others			
				O7b	-Ga3b2$_L$-	O2	106.6(2)
				O7b	-Ga3b2$_L$-	O14b	99.8(2)
				O12d$_L$	-Ga3b2$_L$-	O2	77.2(2)
				O12d$_L$	-Ga3b2$_L$-	O14b	83.5(2)

$O12c_L$	$-Ga3b2_L-$	O2	87.8(2)
$O12c_L$	$-Ga3b2_L-$	O14b	83.0(2)
Average			89.7

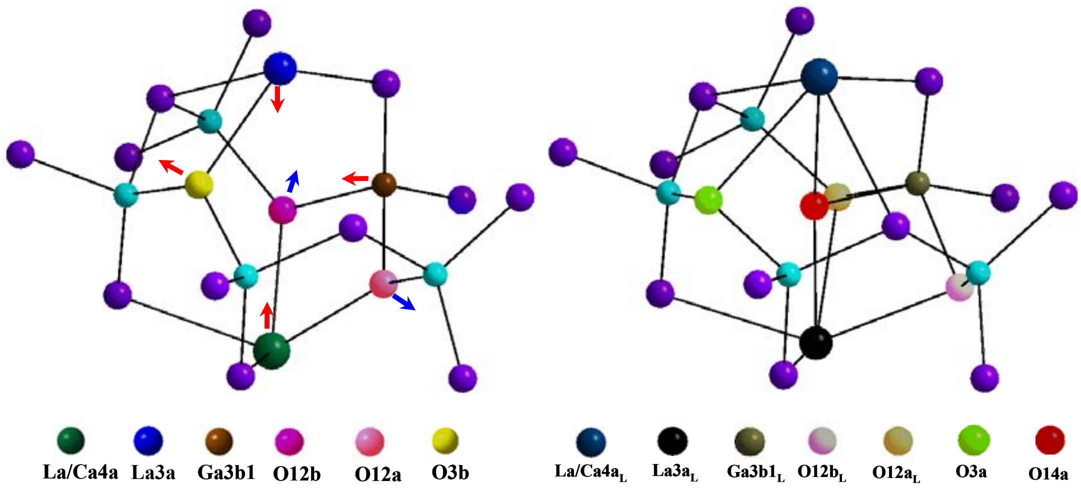

Figure S7.2 Environment of the C_1 ring centroid in (left) bulk and (right) defect structure in $La_{1.64}Ca_{0.36}Ga_3O_{7.32}$. The local displacement of La3a, La/Ca4a, Ga3b1, O12a, and O12b to accommodate the O14a interstitials are marked with arrows in the bulk structure. The displacements (Å) calculated using the split-model for La3a, La/Ca4a, Ga3b1, O12a, and O12b are 0.19(1) ($La3a-La3a_L$), 0.14(1) ($La/Ca4a-La/Ca4a_L$),0.17(1) ($Ga3b1-Ga3b1_L$), 0.56(1) ($O12a-O12a_L$), and 0.49(1) ($O12b-O12b_L$), respectively.

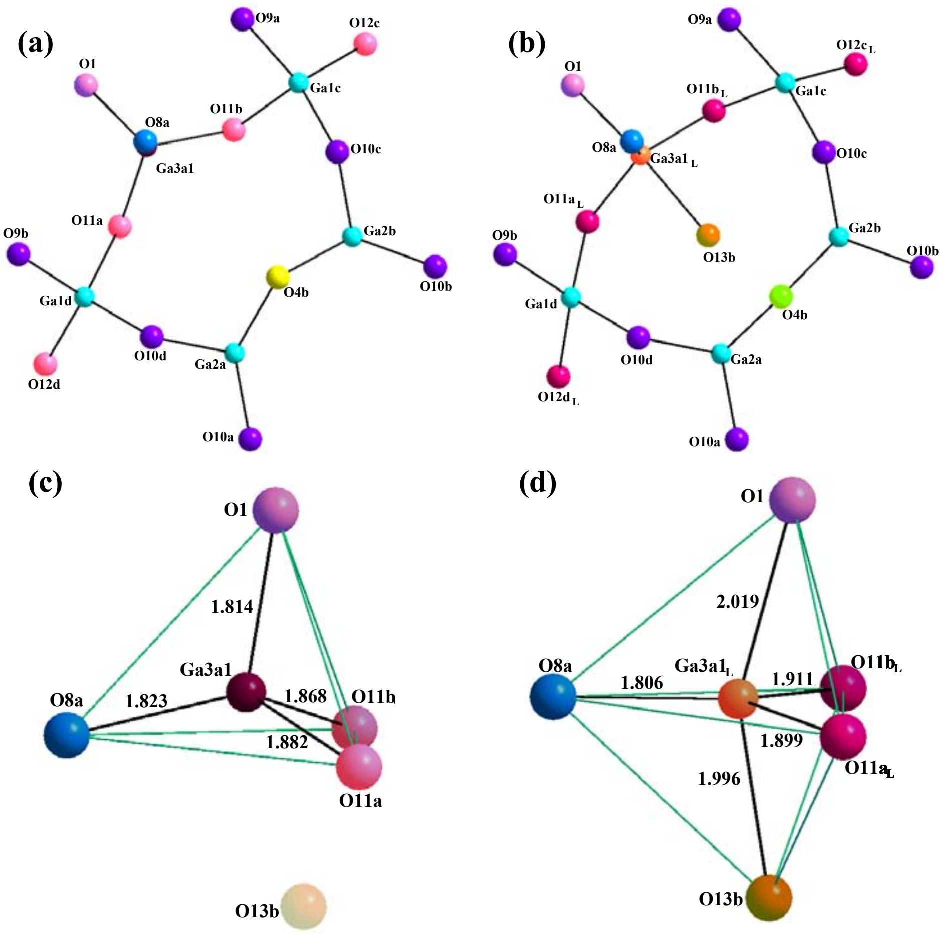

Figure S7.3 Structural relaxation around the interstitial oxide O13b in the split-model of $La_{1.64}Ca_{0.36}Ga_3O_{7.32}$. $Ga3a1_L$, $O11a_L$, $O11b_L$, O4b, and $La3a_S$ are involved in the local structural relaxation. (a) and (c) represent the refined structure in the absence of the interstitial O occupancy of this site with (b) and (d) showing the ring geometry and Ga trigonal bipyramidal structure formed when the interstitials (occupancy of 17.9(1)%) are present. O8a is the terminal oxygen in the polyhedron.

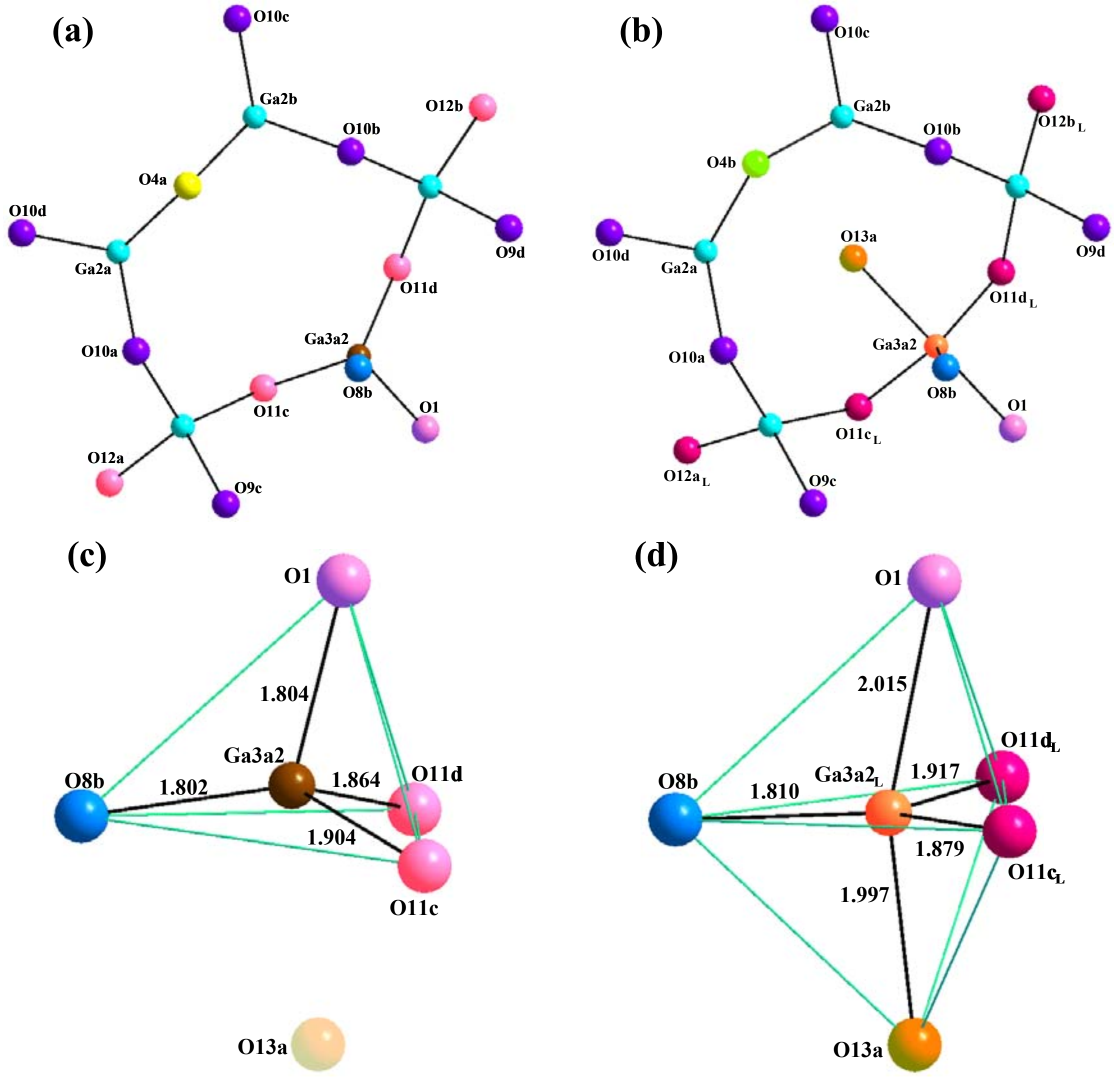

Figure S7.4 Structural relaxation around the interstitial oxide O13a in the split-model of $La_{1.64}Ca_{0.36}Ga_3O_{7.32}$. $Ga3a2_L$, $O11c_L$, $O11d_L$, O4b, $La3b_L$ and $La/Ca4b_L$ are involved in the local structural relaxation. (a) and (c) represent the refined structure in the absence of the interstitial O occupancy of this site with (b) and (d) showing the ring geometry and the Ga trigonal bipyramidal structure formed when the interstitials (occupancy of 28.0(1)%) are present. O8b is the terminal oxygen in the polyhedron.

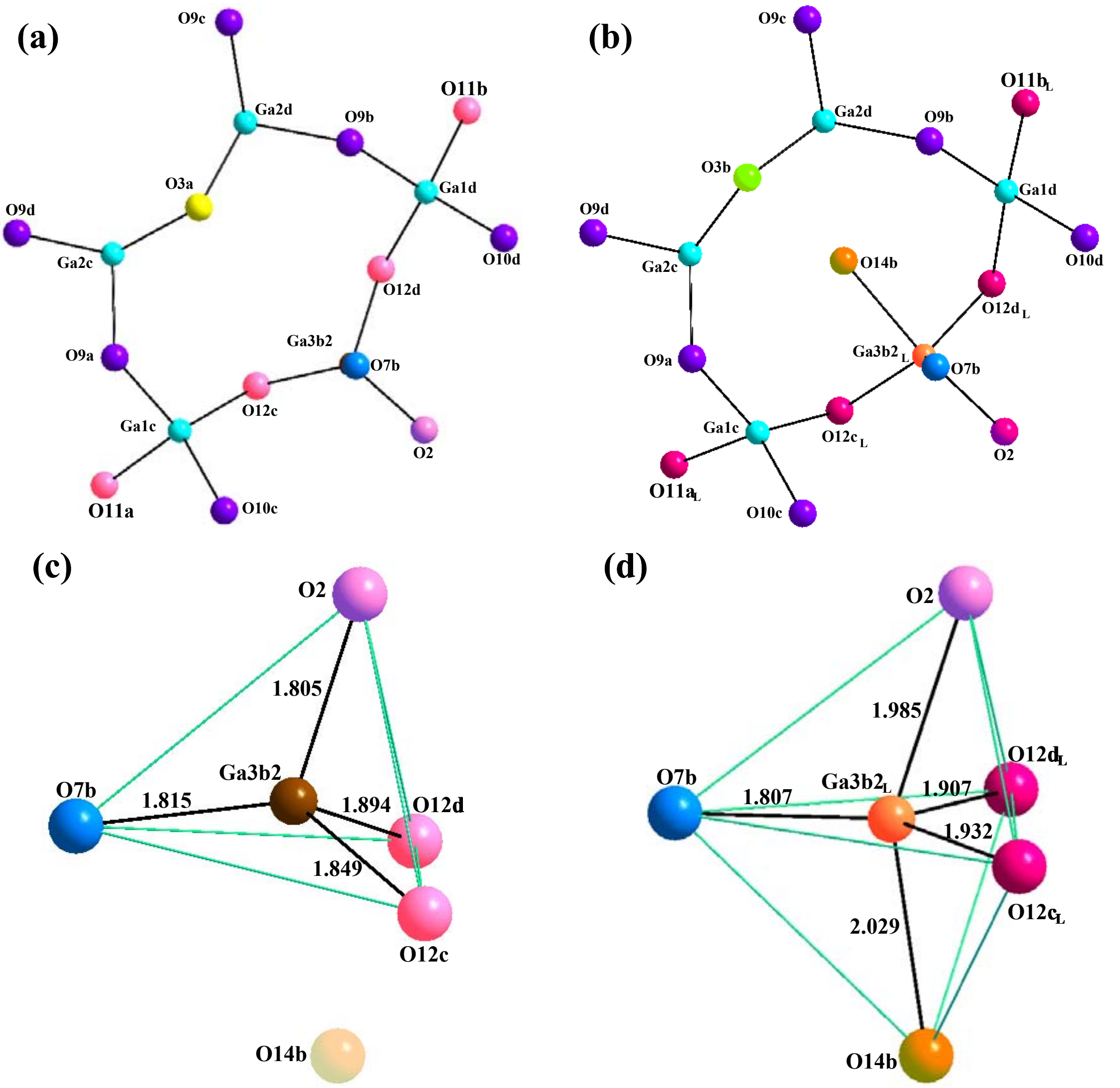

Figure S7.5 Structural relaxation around the interstitial oxide O14b in the split-model of $La_{1.64}Ca_{0.36}Ga_3O_{7.32}$. Ga3b2$_L$, O12c$_L$, O12d$_L$, O3b, and La/Ca4b$_S$ are involved in the local structural relaxation. (a) and (c) represent the refined structure in the absence of the interstitial O occupancy of this site with (b) and (d) showing the ring geometry and Ga trigonal bipyramidal structure formed when the interstitials (occupancy of 23.9(1)%) are present. O7b is the terminal oxygen in the polyhedron.

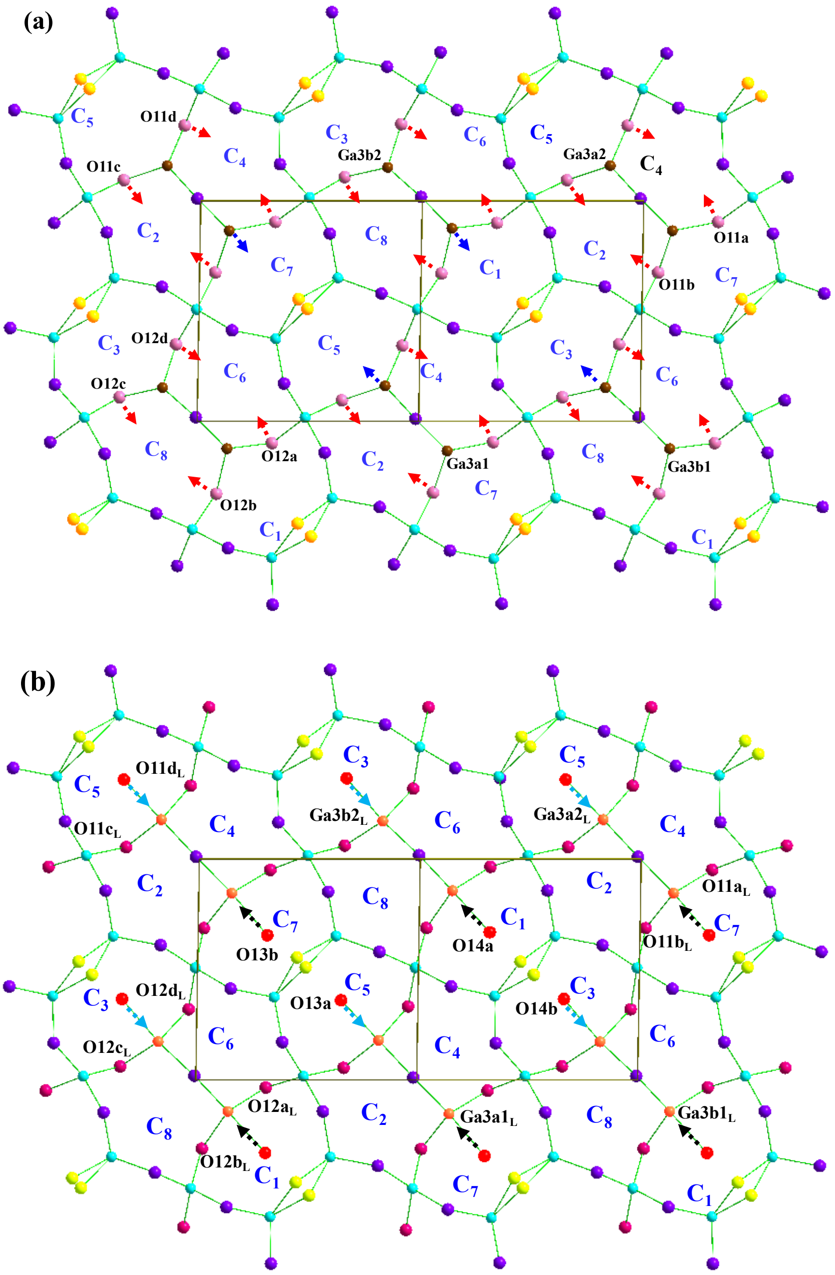

Figure S7.6 Enlarged projection along [110] of (a) bulk and (b) defect structure to show the structural displacement to permit interstitial accomodation. The occupancy of two sets of centroids (C_1/C_7 and C_3/C_5) shares the same direction of displacement as marked by black and cyan arrows in (b), with the sense of the displacement being different in the two pairs.

8. Interstitial oxide and A-site cation ordering--composition and crystal structure

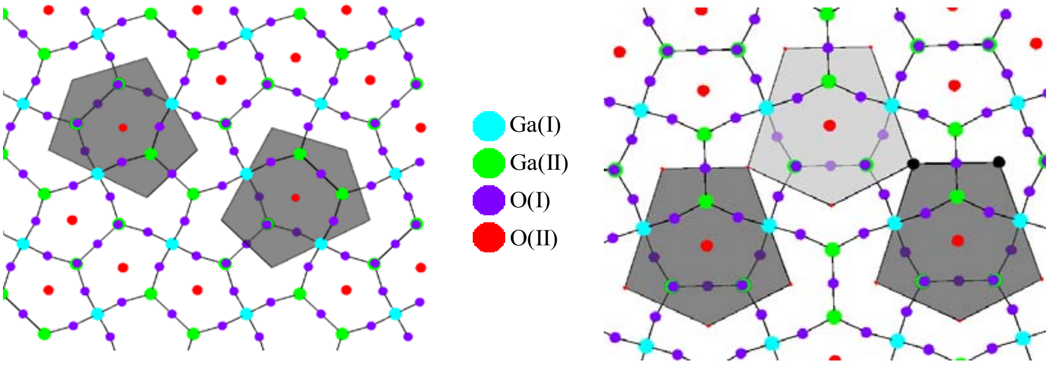

Figure S8.1 Pentagons represent the interstital sites in the five nearest edge-shared rings with a random tiling, corresponding to disordered interstitial occupancy of the 5-rings which are the centroids of the pentagons, with interstitial occupancy > 1/8 (left), and maximally doped structure of either randomly 2/3 occupied or partially ordered by dark grey, fully occupied and light grey 1/3 occupied (right, dark spheres represent two ring centroids sharing a common three-connected Ga_2O_7 edge). $Ga(I)O_4$, 4-connected; $Ga(II)O_4$, 3-connected; O(I), framework oxygens; O(II), interstitial sites.

References

1. Larson, A. C., von Dreele, R. B., General Structure Analysis System (GSAS), Los Alamos National Laboratory Report LAUR 86-748. 2004.

2. P. W. Stephens, J. Appl. Crystallogr. 1999, 32, 281.

3. Coelho, A., *TOPAS-Academic, General Profile and Structure Analysis Software for Powder Diffraction Data*, version 4.1; http://www.topas-academic.net/, 2007.
